# Machine learning approaches for real-time ZIP code and county-level estimation of state-wide infectious disease hospitalizations using local health system data

**DOI:** 10.1016/j.epidem.2025.100823

**Published:** 2025-04-03

**Authors:** Tanvir Ahammed, Md Sakhawat Hossain, Christopher McMahan, Lior Rennert

**Affiliations:** a Department of Public Health Sciences, Clemson University, Clemson, SC, USA; b Center for Public Health Modeling and Response, Clemson University, Clemson, SC, USA; c School of Mathematical and Statistical Sciences, Clemson University, Clemson, SC, USA

**Keywords:** Infectious disease burden, Hospitalization, EHR, Machine learning, Prediction, Nowcasting, COVID-19

## Abstract

The lack of conventional methods of estimating real-time infectious disease burden in granular regions inhibits timely and efficient public health response. Comprehensive data sources (e.g., state health department data) typically needed for such estimation are often limited due to 1) substantial delays in data reporting and 2) lack of geographic granularity in data provided to researchers. Leveraging real-time local health system data presents an opportunity to overcome these challenges. This study evaluates the effectiveness of machine learning and statistical approaches using local health system data to estimate current and previous COVID-19 hospitalizations in South Carolina. Random Forest models demonstrated consistently higher average median percent agreement accuracy compared to generalized linear mixed models for current weekly hospitalizations across 123 ZIP codes (72.29 %, IQR: 63.20–75.62 %) and 28 counties (76.43 %, IQR: 70.33–81.16 %) with sufficient health system coverage. To account for underrepresented populations in health systems, we combined Random Forest models with Classification and Regression Trees (CART) for imputation. The average median percent agreement was 61.02 % (IQR: 51.17–72.29 %) for all ZIP codes and 72.64 % (IQR: 66.13–77.69 %) for all counties. Median percent agreement for cumulative hospitalizations over the previous 6 months was 80.98 % (IQR: 68.99–89.66 %) for all ZIP codes and 81.17 % (IQR: 68.55–91.33 %) for all counties. These findings emphasize the effectiveness of utilizing real-time health system data to estimate infectious disease burden. Moreover, the methodologies developed in this study can be adapted to estimate hospitalizations for other diseases, offering a valuable tool for public health officials to respond swiftly and effectively to various health crises.

## 1. Introduction

The coronavirus 2019 (COVID-19) pandemic caused unmatched difficulties for global public health systems. With over 700 million recorded infections and at least 7 million lives lost worldwide (WorldoMeter, 2024), the extent of its impact is overwhelming. North America, with the highest COVID-19 case fatality rate (Ahammed et al., 2021), has been especially hard hit. This severe impact is evident in the United States, where hospitalizations have already surpassed 6 million by April 13, 2024 (CDC, 2024). In many states, COVID-19 cases filled hospitals to nearly total capacity at the peak. Frontline healthcare workers in 60–75 % of hospitals were exhausted from the unexpected and heavy COVID-19 workload, leading to a higher death toll (Li et al., 2023). Even during the week of April 7 to April 13, 2024, 6406 patients from various states were hospitalized due to COVID-19 (CDC, 2024). Thus, an accurate representation of COVID-19 hospitalizations is crucial. These data insights will enable healthcare authorities to allocate resources efficiently, such as increasing ICU capacity or redistributing medical staff to high-impact areas (e.g., mobile health clinics) (Gezer et al., 2024). Policymakers and healthcare administrators can use these estimates of COVID-19 hospitalizations to allocate hospital beds, medical supplies, and staffing more effectively (Ma and Rennert, 2024). Additionally, the data help identify hotspots requiring immediate intervention, support public health planning, and guide the implementation of preventive measures like localized lockdowns and vaccination campaigns. The insights facilitate proactive rather than reactive responses, ultimately enhancing the overall management of the pandemic. Creating a framework that accurately estimates COVID-19 hospitalizations can also be invaluable for future epidemics or pandemics caused by other infectious diseases or variants of COVID-19.

There are currently few techniques available for estimating COVID-19 hospitalizations (Couture et al., 2022). For example, Reese et al. calculated COVID-19 hospitalizations by adjusting confirmed case counts to account for under-detection. They used a probabilistic multi-plier model, adjusting nationally reported laboratory-confirmed case counts for under-detection based on testing practices in inpatient and outpatient settings and assay sensitivity (Reese et al., 2021). Other studies have used seroprevalence surveys to estimate the number of COVID-19 hospitalizations (Hozé et al., 2021; Angulo et al., 2021). Couture et al. used surveillance data from the COVID-19-Associated Hospitalization Surveillance Network (COVID-NET) and a Bayesian hierarchical model to estimate monthly COVID-19 hospitalization rates for all 50 states (Couture et al., 2022).

Many prediction techniques have also been assessed so far. The most common among these are compartmental models and machine learning techniques (Wang et al., 2022). For instance, Kinsey et al. developed improved SEIR models to capture COVID-19 dynamics and forecast hospitalizations by incorporating external factors such as spatial-temporal and mobility data (Kinsey et al., 2024). Jin et al. used attention and transformer models to analyze and combine previous COVID-19 trends to make future predictions (Jin et al., 2021). Rodríguez et al. used a neural network architecture with COVID-19 time series and mobility data as inputs (Rodríguez et al., 2021). Endres-Dighe et al. discussed the development of a statewide simulation model for predicting COVID-19’s impact on healthcare resources and capacity in North Carolina using a geospatially explicit healthcare facility network model (Endres-Dighe et al., 2021). Wang et al. combined Google search information and COVID-19 related time series information with dynamic training and rolling window prediction for forecasting future 2 weeks of national and state-level COVID-19 new hospital admissions in the United States (Wang et al., 2022). Wesner et al. proposed a Bayesian non-linear regression model based on the Weibull function to forecast cumulative and active COVID-19 hospitalizations in South Dakota, USA (Wesner et al., 2021).

The estimation or prediction of COVID-19 hospitalizations has primarily been conducted at the state or national level (Li et al., 2023; Yaesoubi et al., 2023). To address this, Yaesoubi et al. developed a framework for creating simple classification rules to predict whether COVID-19 hospitalizations will exceed local capacity within four or eight weeks (Yaesoubi et al., 2023). Similarly, Li et al. predicted weekly new COVID-19 hospitalizations in 159 counties across the USA using Random Forest models on 20 months of wastewater surveillance data (Li et al., 2023). To estimate hospitalizations in Ohio, KhudaBukhsh et al. projected statewide estimates of COVID-19 incidence over time and incorporated a geographic component to translate the statewide model’s output into hospital burden estimates for each county or "hospital catchment" area (KhudaBukhsh et al., 2023). In another study (Gao et al., 2023), the authors proposed a model called HOIST for COVID-19 hospitalizations prediction. HOIST leverages Ising dynamics, a statistical physics concept, to extract and utilize spatial relationships across different locations. The model was evaluated using rich linked data-bases, including insurance claims, census information, and hospital resource usage data across 2299 counties in the USA.

These forecasts depend on historical data on daily or weekly COVID-19-induced hospitalizations or verified COVID-19 cases as primary indicators (Li et al., 2023). Conventional methods of obtaining comprehensive state-wide data for estimating COVID-19 hospitalizations have limitations, particularly when time is of the essence, such as the early stages of an epidemic or the emergence of novel variants. Real-time data is crucial for effective responses. Yet, bureaucratic processes and logistical issues often slow down data collection and access, making it hard to get timely information. This delay affects the development of precise estimation models, slowing down public health responses. In many cases, local health systems or hospitals can provide timely and granular data compared to state health department data. However, health system data is not representative of all regions. For example, Prisma Health, which is the largest non-for-profit health system in South Carolina (SC), covers approximately 25 % of the state’s population (Prisma Health, 2024). Because of this geographic restriction, imputing hospitalization estimates for populations underrepresented in healthcare systems is essential for estimating hospitalizations using local health system data.

Unfortunately, estimates and forecasts of infectious disease burden at the county level are not always useful for directing field-level interventions (Borquez and Martin, 2022; Rennert et al., 2024). For example, mobile health clinics (MHCs) deliver health care to medically underserved communities. While county-level forecasts may yield higher accuracy, there is substantial variation among ZIP codes within a county (Gezer et al., 2024). Previous studies have shown that targeting local communities based on disease burden can improve the effectiveness of MHC in reaching more high-risk individuals (Rennert et al., 2024; Howard et al., 2024). However, models targeting more granular geographic regions (e.g., ZIP codes), which are necessary to inform effective field-level interventions such as MHCs (Gezer et al., 2024), have received little to no attention for estimating disease burden from respiratory infectious diseases.

To the best of our knowledge, there is currently little to no work on models that must rely on the use of partial data (i.e., unrepresentative health system data) to estimate real-time infectious disease burden for an entire state, let alone for granular geographic regions where no data exists. The primary objective of this study is to develop a novel estimation framework for all COVID-19 hospitalizations at the ZIP code and county level, utilizing only partial data obtained from the electronic health records (EHR) of a local health system. The approach we present here is most useful in situations where limited electronic health records are the only source of real-time data (e.g., health system data) for modelers. We demonstrate that our approach can reasonably estimate infectious disease burden in regions with partial data as well as regions with no data.

## 2. Methodology

### 2.1. Overview

We employed two approaches to estimate infectious disease burden. The first was to estimate the number of hospitalizations in each region over the previous six months. These estimates were then compared to observed hospitalizations in the same regions obtained from the South Carolina Office of Revenue and Fiscal Affairs (SC RFA) data. There is approximately a 6-month lag between the date of the encounter and the time when the SC RFA obtains the corresponding claim data due to the delays in data reporting. Additionally, there is an expected delay of two to four weeks following the researchers’ request for data from the SC RFA. Such information is useful for future planning. For example, Clemson Rural Health (CRH) uses this information to identify high-risk areas where they do not currently operate. While this retroactive information does not allow for immediate allocation of MHCs, it allows CRH to develop community partnerships in new areas where they do not currently serve. This is necessary for delivering an MHC (or other field-level interventions) to communities (Rennert et al., 2024). The second approach was to estimate the number of current hospitalizations (weekly nowcasting). This directly supports real-time decision-making related to the delivery of MHCs to high-risk communities.

We employed a retrospective observational design to estimate state-wide COVID-19 hospitalizations using data collected from the Prisma Health system. Prisma Health is SC’s largest not-for-profit healthcare provider, serving over 1.2 million patients annually. Prisma Health’s COVID-19 data registry was developed during the pandemic and has been used to evaluate factors associated with COVID-19 severity (Schockling et al., 2020). Data received from Prisma Health is lagged by 24 hours.

The steps for developing an estimation framework for COVID-19 hospitalizations were as follows:

1. Estimating hospitalizations in areas with strong Prisma Health coverage:

Initially, we identified areas with robust Prisma Health coverage and estimated state-wide hospitalizations using data from Prisma Health. These estimates were then compared to observed hospitalizations in the same regions obtained from the SC RFA data. We used negative binomial generalized linear mixed models and Random Forest models to estimate hospitalizations.

2. Imputing hospitalizations in areas with limited or no Prisma Health coverage:

We imputed hospitalization estimates for regions with limited or no Prisma Health coverage based on our initial estimates. The imputation utilized advanced techniques, such as the Random Forest algorithm, Predictive Mean Matching (PMM), Classification and Regression Trees (CART), and Lasso Linear Regression methods. These imputed values were then compared with observed hospitalizations recorded by the SC RFA for those areas to validate the accuracy of our imputation techniques.

3. Evaluate overall performance:

Finally, we integrated the estimated hospitalizations for areas with strong Prisma Health coverage and the imputed values for regions with limited or no coverage to create a comprehensive dataset. This dataset was then compared with the state-wide observed hospitalizations recorded in the SC RFA dataset to assess the overall accuracy of our estimation framework.

### 2.2. Data sources

The hospitalization data were obtained from the Prisma Health COVID-19 registry and the South Carolina Office of Revenue and Fiscal Affairs (SC RFA) (South Carolina Revenue and Fiscal Affairs Office, 2024). ICD-10 codes for COVID-19 hospitalization obtained from Prisma Health and SC RFA are provided in Supplementary Tables S1 and S2, respectively.

Age, race, sex, home ZIP code, and county were included as individual-level variables. Demographic variables, linked to each hospitalized patient’s ZIP code or county, were sourced from the United States Census Bureau. These variables were the percentages of age, sex, and race groups and the total population size within each ZIP code or county.

### 2.3. Study settings

The study sample included individuals of all ages hospitalized due to COVID-19 between March 2, 2020, and December 31, 2023. To improve our models’ statistical power and accuracy, we narrowed our analysis to SC counties and ZIP codes whose total hospitalization numbers recorded by Prisma Health were above a predetermined threshold (50 for ZIP codes, 100 for counties) during the study period (March 2, 2020, to December 31, 2023).

Predictor variables included sex, age, race, total hospitalizations recorded by Prisma Health, and the log-transformed sub-population size within each ZIP code or county. We calculated the subpopulation size for each demographic group by multiplying the total population by the respective percentages of gender, age group, and race group. The term "number of hospitalizations" refers to the number of unique individuals hospitalized within the specified time interval.

### 2.4. Analysis

#### 2.4.1. Estimating retroactive statewide hospitalizations using health system EHR in regions with sufficient Prisma Health coverage

We used negative binomial and Random Forest models to estimate the number of hospitalizations in each ZIP code and county with sufficient representation in the Prisma Health System. This is defined as at least 50 hospitalizations per ZIP code (N = 125) and 100 hospitalizations per county (N = 19). There are 424 ZIP codes and 46 counties in SC. Therefore, we have sufficient coverage (per our definition) for 29.5 % of ZIP codes and 41.3 % of counties in SC.

##### 2.4.1.1. Negative binomial models.

The negative binomial models used have the following forms:

1. $\log\left(\mu^{H_{i,s}^{RFA}}\right)∼\beta_{0}+\beta_{1}\times H_{i,s}^{PH}+\beta_{2}\times \log(subpopulation\;size_{s})+b_{i}$
2. $\log\left(\mu^{H_{i,s}^{RFA}}\right)∼\beta_{0}+\beta_{1}\times I_{Age,s=20-44}+\ldots +\beta_{3}\times I_{Age,s=65+}+\beta_{4}\times I_{race,s=white}+\;\;\beta_{5}\times \;\;I_{sex,s=M}+\;\;\beta_{6}\times \;\;H_{i,s}^{PH}+\;\;\beta_{7}\times \log(subpopulation\;size_{s})+b_{i}$
3. $\log\left(\mu^{H_{i,s}^{RFA}}\right)∼\beta_{0}+\beta_{1}\times I_{Age,s=20-44}+\ldots +\beta_{3}\times I_{Age,s=65+}+\beta_{4}\times I_{race,s=white}+\beta_{5}\times I_{sex,s=M}+\beta_{6}\times H_{i,s}^{PH}+\beta_{7}\times H_{i,s}^{PH}\times I_{Age,s=20-44}+\ldots +\beta_{11}\times H_{i,s}^{PH}\times I_{sex,s=M}+\beta_{12}\times \log(subpopulation\;size_{s})+b_{i}$
4. $\log\left(\mu^{H_{i,s}^{RFA}}\right)∼\beta_{0}+\beta_{1}\times I_{Age,s=20-44}+\ldots +\beta_{3}\times I_{Age,s=65+}+\beta_{4}\times I_{race,s=white}+\beta_{5}\times I_{sex,s=M}+\beta_{6}\times H_{i,s}^{PH}+\beta_{7}\times \log(subpopulation\;size_{s})$
5. For each ZIP code/county i, $\Delta_{i}=\mu^{H_{RFA,total;i}}-\mu^{H_{PH,total;i}}$

$$g(\Delta_{i})∼\beta_{0}+\beta_{1}\times I_{Age,s=20-44}+\ldots +\beta_{3}\times I_{Age,s=65+}+\beta_{4}\times I_{race,s=white}+\beta_{5}\;\;\times I_{sex,s=M}+\beta_{6}\times \log(subpopulation\;size_{s})+b_{i}$$

The outcome variable $H_{i,s}^{RFA}$ represented the number of COVID-19 hospitalizations recorded in the i-th ZIP code or county for the s-th sub-group, as documented by the SC RFA. Similarly, $H_{i,s}^{PH}$ denoted the number of COVID-19 hospitalizations documented by Prisma Health in the i-th ZIP code or county for the s-th sub-group. $\mu^{H_{i,s}^{RFA}}$ is the conditional mean of $H_{i,s}^{RFA}$ given the covariates. $b_{i}$ were the random effects for intercept and were included for each ZIP code or county and assumed to follow a normal distribution.

We used the ‘glmer.nb’ function from the ‘lme4’ package in R for the negative binomial models. In ‘glmer.nb’, the dispersion is managed by estimating an additional dispersion parameter θ, allowing the variance to exceed the mean, which accounts for overdispersion.

##### 2.4.1.2. Random Forest models.

The Random Forest algorithm is a robust machine learning technique designed to manage non-linear data tendencies effectively. In this study, we developed two different Random Forest models to estimate the number of hospitalizations recorded by the SC RFA using Prisma Health data. In Model 1, we used the number of hospitalizations recorded by Prisma Health and the log-transformed subpopulation size. For a more comprehensive model, we included additional demographic variables: sex, age group, and race group in Model 2.

We used the ‘caret’ package in R to train and optimize our Random Forest models. We defined the control parameters for our model training using 5-fold cross-validation. To improve the model, we conducted a grid search to find the optimal value of ‘mtry,’ which is the number of variables randomly sampled as candidates at each split. We used ‘ntree’ (the number of trees in the forest) = 300 for our models. To ensure that the models were not overly complex, we used the default values for ‘nodesize’ (the minimum size of terminal nodes) and ‘maxnodes’ (the maximum number of terminal nodes) for both models.

##### 2.4.1.3. Accuracy of the models.

To evaluate the accuracies of the models, we separated the data into training and testing sets. The estimated hospitalizations were then compared to the observations in the testing set. The training set consisted of data collected between January 1, 2023, and June 30, 2023, while the testing set spanned from July 1 to December 31, 2023.

The total number of hospitalizations for each area i was aggregated as follows:

$$\sum_{subpopulations=1}^{s=n}H{\text{ospitalizations}}_{i,s}$$

For each ZIP code or county i, the percentage agreement accuracy was calculated as,

$$A_{i}=\frac{\text{min}(O_{i},\;P_{i})}{\max(O_{i},\;P_{i})}$$

where $O_{i}$ and $P_{i}$ were the total observed and estimated hospitalizations over the estimation period, respectively.

To check the robustness of our framework, we followed the same procedure using a training set consisting of data collected between December 1, 2020, and June 30, 2021, while the testing set spanned from July 1 to December 31, 2021.

#### 2.4.2. Imputation of statewide hospitalizations in regions with insufficient Prisma Health coverage

In the majority of ZIP codes (70.5 %), Prisma Health data was not available. Due to heterogeneity in health system coverage within a region, it is not possible to predict what health system-level hospitalizations would be without knowing the size of that health system. So, it was not possible to estimate the hospitalizations for those ZIP codes or counties using the number of hospitalizations reported in Prisma Health as a predictor variable. Therefore, we used demographic and socioeconomic community characteristics to estimate what the total number of hospitalizations should be in that geographic region (which is our outcome of interest, as opposed to the subset of hospitalizations from a particular health system). In this case, there were 125 ZIP codes with sufficient Prisma Health coverage (as defined by at least 50 COVID-19 hospitalization encounters from March 2, 2020, to December 31, 2023). We imputed the hospitalization estimates for the areas with insufficient or no Prisma Health coverage. At the ZIP code level, we imputed hospitalizations for 180 ZIP codes. South Carolina has 424 ZIP codes, but we excluded certain ones due to a lack of state-level hospitalization data. Specifically, 119 ZIP codes reported no hospitalizations from July to December 2023, which prevented us from validating the imputed estimates for these areas. These ZIP codes, which represent 2.7 % of South Carolina’s population, typically correspond to sparsely populated areas, such as forests, or large institutions like universities and commercial districts with minimal residential populations. However, others may lack data due to their small population sizes (Gezer et al., 2024). Consequently, only ZIP codes with matching state-level hospitalization data but underrepresented in Prisma Health were imputed (Supplementary Fig. S1).

We utilized both the ‘mice’ and ‘missForest’ packages in R to impute the estimates. ‘mice’ operates on the principle of creating multiple imputed datasets based on the Multivariate Imputation by Chained Equations (MICE) technique. It was used to handle missing data by iteratively imputing values based on the estimates generated by the models in regions with sufficient health system data. It begins by filling missing values with initial guesses, typically the observed data’s mean or median. Then, it applies a chained equation approach, where each variable with missing data is imputed sequentially using regression models based on other variables. This process is repeated for multiple iterations (5 iterations for this imputation process), creating several imputed datasets. Each dataset undergoes statistical analysis (e.g., regression), and the results are combined by averaging the estimates across the imputed datasets to produce overall estimates. The associated variances are calculated considering both within-imputation and between-imputation variance (Wulff and Ejlskov, 2017). We used “pmm” (Predictive Mean Matching), “cart” (Classification and Regression Trees), and “lasso.norm” (Lasso Linear Regression) methods from the ‘mice’ package. PMM predicts missing values based on observed values from similar cases, ensuring that imputed values are plausible within the context of the data distribution. CART constructs a decision tree to model the relationships between variables, making it suitable for handling non-linear relationships and interactions in the data. Lasso Linear Regression employs Lasso regularization to enhance prediction accuracy by selecting a subset of predictor variables, effectively reducing the model’s complexity. Here, we used individual-level demographic variables (e.g., sex, age, race), the log-transformed population for each sub-category, and community-level demographic data, which is population distribution by age, gender, and race (e.g., percent of female, percent of white) at that ZIP code or county for imputing prediction of hospitalizations in regions without sufficient data. In applying the MICE method, it is important to note that the process does not involve a conventional training phase akin to those employed in supervised machine learning models. Instead, MICE relies on using observed data to impute missing values. On the other hand, the ‘missForest’ imputation technique is based on the Random Forest algorithm, a non-parametric method. At each step, Random Forest models were built to predict the missing predicted hospitalizations using the other variables as predictors.

Following the imputation process, we re-aggregated each area’s total number of hospitalizations and recalculated the percentage agreement between the observed and estimated hospitalization numbers to assess the accuracy of our imputation.

#### 2.4.3. Weekly nowcasting

For weekly estimation, the training set included data collected until July 2nd, 2023, while the testing set covered July 3rd to October 1st, 2023. This period corresponds to the last available wave represented in the SC RFA data (which is used to validate the framework).

The week of observation, the total weekly hospitalizations reported in Prisma Health for each county or ZIP code, the log-transformed total population for that county or ZIP code, and the county or ZIP code identifiers were used as predictor variables in the Random Forest model. The total hospitalizations for each week were not subdivided into demographic categories such as sex, age, and race groups, as doing so would result in many instances of zero hospitalizations due to the smaller sample sizes within each subgroup.

For each ZIP code or county i, the weekly average percentage agreement accuracy was calculated as,

$$\text{weekly percentage agreement accuracy},\;A_{i,w}=\frac{\text{min}(O_{i,w},\;P_{i,w})}{\max(O_{i,w},\;P_{i,w})}\;\text{average weekly percentage agreement accuracy:}\;\frac{\sum_{w=1}^{w=n}A_{i,w}}{n}$$

where $O_{i,w}$ and $P_{i,w}$ were the total observed and estimated hospitalizations over each week w, respectively.

The `mice` package with the CART (Classification and Regression Trees) method was applied to impute the missing predicted hospitalizations in areas with limited or no Prisma Health coverage. The imputation process used predictors, including county or ZIP code, Week, demographic characteristics such as population distribution by age, gender, and race, and the log-transformed total population for each county or ZIP code.

## 3. Results

The demographic characteristics of the hospitalized participants for selected ZIP codes and counties were summarized in Tables 1 and 2. A similar demographic distribution between the Prisma Health and SC RFA datasets was observed, with slight differences. In both datasets, hospitalized participants were predominantly female and middle-aged or older adults. The Prisma Health system had a higher representation of White individuals (ZIP code level: 64.24 % and county level: 64.15 %) than the SC RFA across these selected areas. The SC RFA system captured more youths than Prisma Health, both at the ZIP code (21.09 % vs 24.71 %) and county levels (21.15 % vs 23.97 %). Conversely, Prisma Health data showed a higher percentage of participants aged 65 and above (31.30 % at the ZIP code level) than SC RFA (26.54 %). This indicated that Prisma Health represented older individuals in the selected areas more than the SC RFA system.

**Fig. 1.**
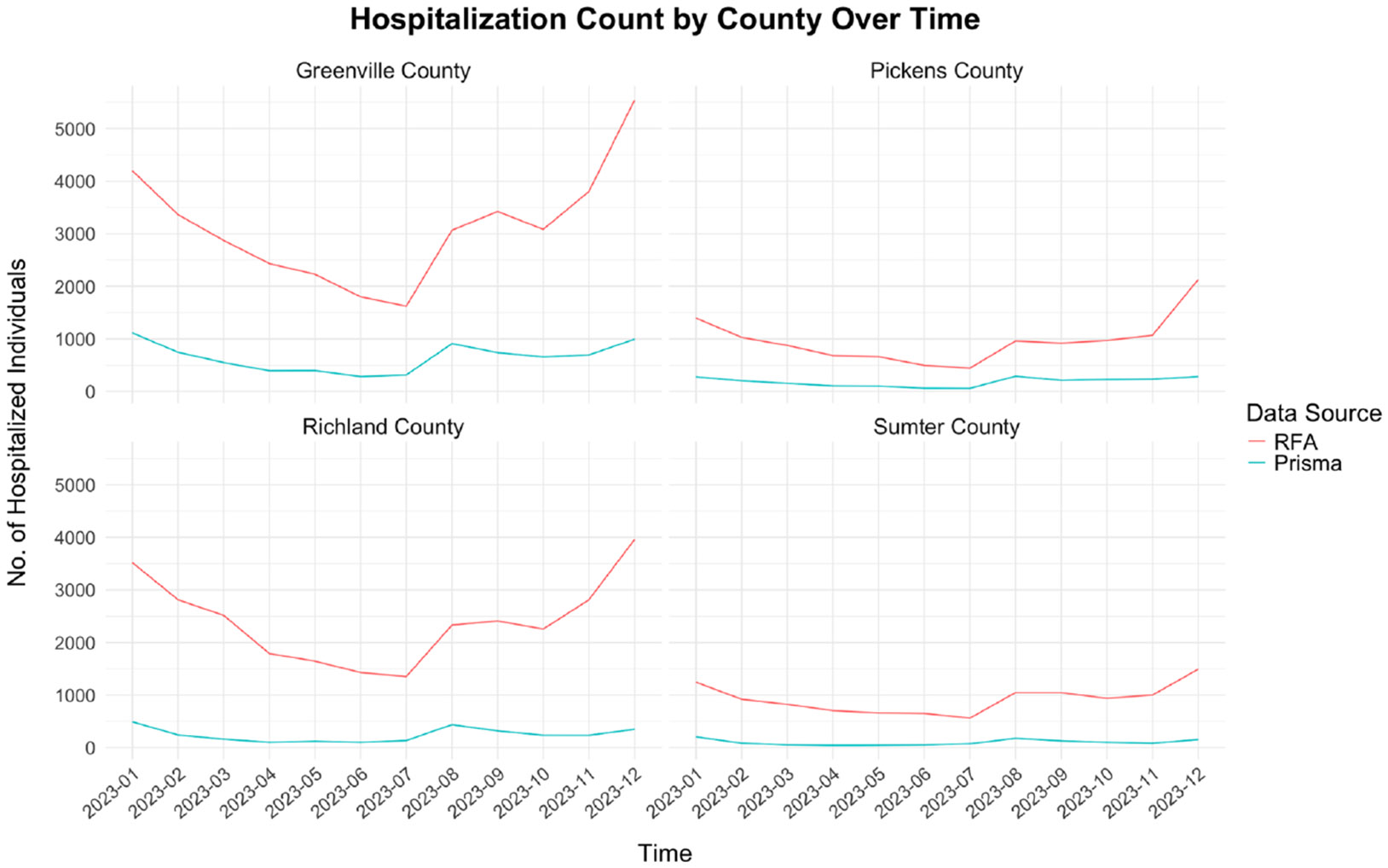
Total observed hospitalization counts for Greenville, Richland, Sumter, and Pickens counties based on Prisma Health and statewide data (obtained via SC RFA) from “2023-31-01” to "2023-12-31.”.

Fig. 1 presents the distribution of total observed hospitalizations, comparing data from Prisma Health and the SC RFA. As an example, we only presented Greenville, Richland, Sumter, and Pickens counties in this figure. Both data sources exhibited similar overall trends, with consistent relative increases and decreases, indicating comparable patterns despite differences in absolute hospitalization counts. Notable peaks in hospitalization counts were observed around December-January and August-September.

### 3.1. Retrospective 6-months estimations

Table 3 displays the median percent agreement accuracy of estimated hospitalizations across 125 ZIP codes and 19 counties (with sufficient Prisma Health coverage) utilizing EHR from Prisma Health. The table compares the performance of five negative binomial models and two Random Forest models. Supplementary Table S3 and S4 show the total number of observed and estimated hospitalizations across 125 ZIP codes and 19 counties, respectively.

For ZIP code level estimation, the results indicate that the negative binomial Model 2 achieved the highest median accuracy of 85.44 % (IQR: 78.19–91.74 %). This was closely followed by the Random Forest Model 2, which incorporated additional demographic variables, achieving a median accuracy of 85.02 % (IQR: 76.15–93.17 %). To assess the robustness of our framework, we utilized 2021 data for further evaluation. In this analysis, Random Forest Model 2 outperformed the other models with a median accuracy of 86.07 % (IQR: 77.41–92.56 %). In contrast, the negative binomial Model 2 demonstrated a lower median accuracy of 83.68 % (IQR: 76.27–89.89 %), highlighting a significant decrease in its performance relative to the Random Forest model (Supplementary Table S5).

The Random Forest Model 2 is recommended here for hospitalization estimation because of its reliability and consistently high accuracy. It demonstrated resilience to changes in the data. While the Negative Binomial Model 2 initially had a slightly higher accuracy, it was less accurate on the 2021 data, indicating limited generalizability.

For county-level estimation, Random Forest models generally outperformed negative binomial models in estimating hospitalizations. Random Forest Model 2 achieved the highest median accuracy of 88.33 % (IQR: 81.94–93.01 %). The second highest accuracy was achieved by Random Forest Model 1, which incorporated the number of hospitalizations recorded by Prisma Health and the log-transformed subpopulation as the predictors. However, Random Forest Model 1 achieved the highest median accuracy of 89.42 % (IQR: 80.64–92.10 %) for the 2021 dataset (Supplementary Table S6).

**Fig. 2.**
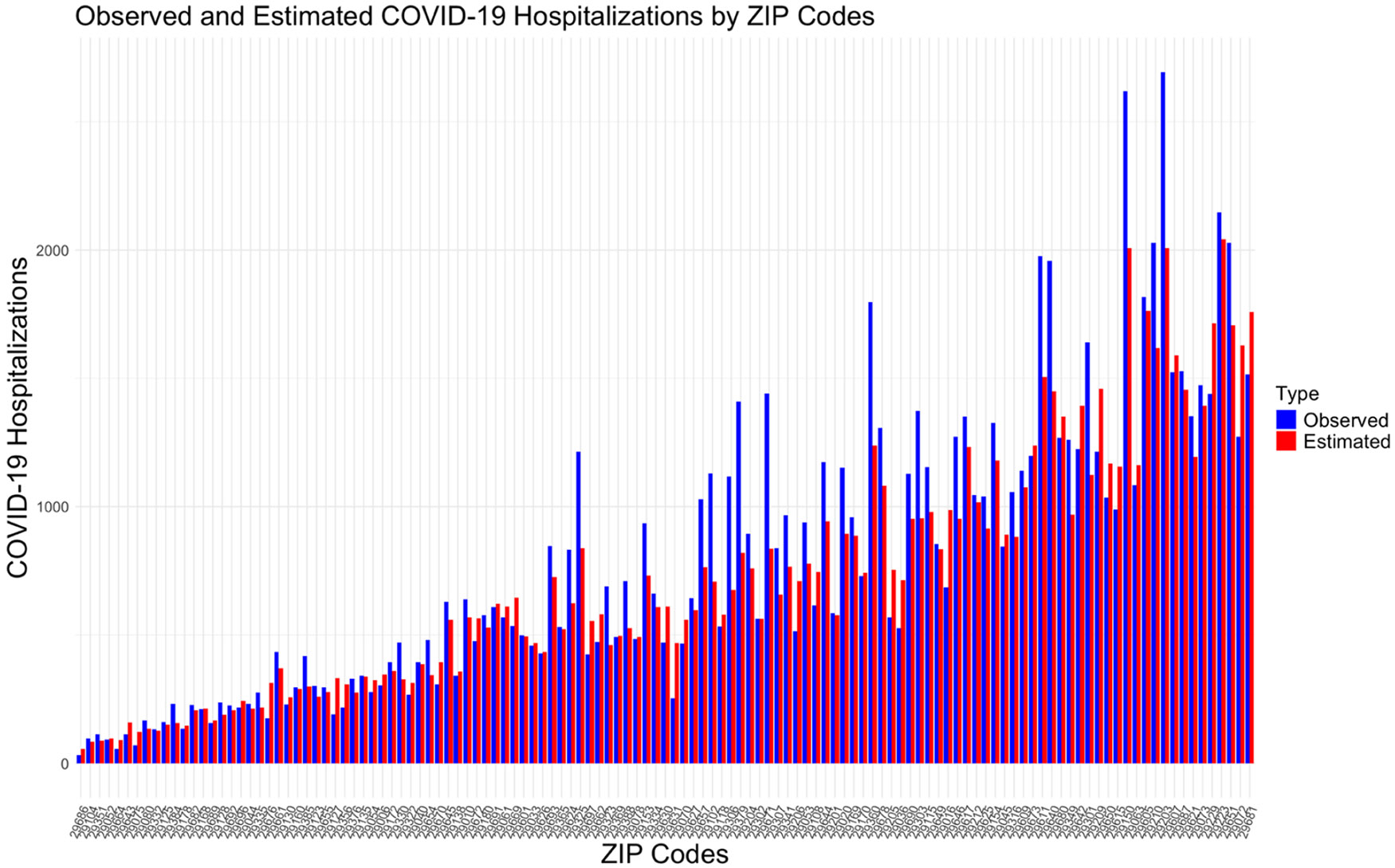
Comparison of observed and estimated COVID-19 cumulative hospitalizations (using Random Forest Model 2) over the previous 6 months (Jul–Dec ’23) in 125 ZIP codes with sufficient Prisma Health coverage (defined by at least 50 COVID-19 hospitalization encounters per ZIP code between 2020 and 2023).

Fig. 2 compares the observed and estimated COVID-19 hospitalizations across those 125 ZIP codes. The estimated hospitalizations used in Fig. 2 were calculated using Random Forest Model 2, which consistently showed higher accuracy across analyses. The ZIP codes were ordered according to their respective populations. The figure shows that, for most ZIP codes, the observed and estimated hospitalization counts closely align. However, the differences between observed and estimated hospitalizations are more pronounced in ZIP codes with larger populations, resulting in higher observed hospitalization counts.

**Fig. 3.**
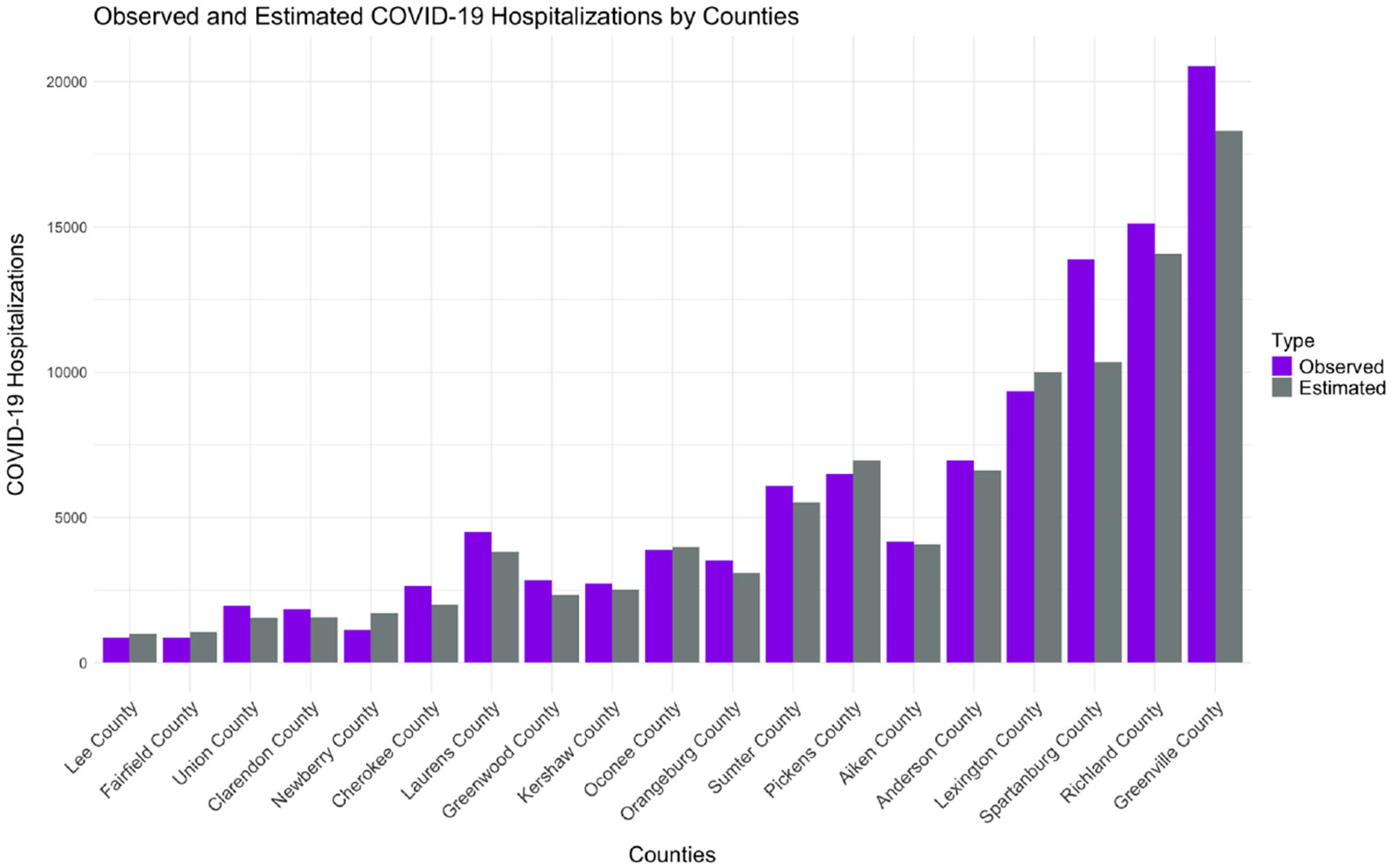
Comparison of observed and estimated (using Random Forest Model 2) COVID-19 cumulative hospitalizations over the previous 6 months (Jul–Dec ’23) in 19 counties with sufficient Prisma Health coverage (defined by at least 100 hospitalizations per county between 2020 and 2023).

Fig. 3 compares the observed and estimated COVID-19 hospitalizations (using Random Forest Model 2) across different counties (ordered according to their respective populations). The figure highlights that the observed and estimated hospitalization counts closely align for most counties. However, similar to the ZIP code levels, differences between the observed and estimated values become more noticeable in counties with higher actual hospitalization counts.

Table 4 illustrates the median percent agreement accuracy of imputed hospitalization estimates across 180 ZIP codes and 27 counties (based on initial estimations in 125 ZIP codes and 19 counties) where Prisma Health coverage is insufficient. The table compares the performance of four imputation methods: Random Forest Algorithm, PMM, CART, and Lasso Linear Regression.

The results in Table 4 indicate that the CART method achieved the highest median accuracy of imputed hospitalizations. The accuracy was 77.13 % (IQR: 60.91–86.22 %) when the estimated hospitalizations used for imputation came from Random Forest Model 2 and 71.35 % (IQR: 56.66–86.57 %) for Negative Binomial Model 2 in imputing for 180 ZIP codes. Similarly, when we followed the same procedure using 2021 data, Random Forest Model 2 combined with CART yielded the highest median accuracy of 73.14 % (IQR: 60.82–83.95 %), followed by CART for Negative Binomial Model 2, achieving a median accuracy of 71.88 % (IQR: 58.18–86.16 %) in 261 ZIP codes (Supplementary Table S7).

Similarly to the ZIP code level estimations, we extended our analysis to the counties where Prisma Health coverage is insufficient and imputed estimates for these additional 27 counties and compared with the observed hospitalizations. We compared Random Forest models because Random Forest models outperformed negative binomial models in estimating county-level hospitalizations (Table 3). Hospitalization estimates imputed through the CART method based on the estimates from Random Forest Model 2 also yielded the highest accuracy of 71.50 % (IQR: 53.21–85.09 %). The second-highest accuracy, 67.43 % (IQR: 53.79–88.56 %), was achieved for estimates imputed through the CART method using the estimates from Random Forest Model 1. Similarly, for county-level imputation using the 2021 dataset, Random Forest Model 2 provided the highest accuracy at 77.96 % (IQR: 56.59–91.88 %) (Supplementary Table S8).

Table 5 presents the median percent agreement accuracy of hospitalization estimates across 305 ZIP codes (combining the 125 ZIP codes with sufficient Prisma Health coverage and the 180 ZIP codes with imputed hospitalizations) and all 46 counties (combining the 19 counties with sufficient Prisma Health coverage and the 27 counties with imputed estimates).

For ZIP codes, the results show that hospitalization estimates imputed through the CART method using the estimates from Random Forest Model 2 provided the highest accuracy of 80.98 % (IQR: 68.99-89.66 %), closely followed by hospitalization estimates imputed through the CART method using the estimates from Negative Binomial Model 2 (79.55 %; IQR: 66.86–89.89 %). For 2021 data, we also observed the same (Random Forest Model 2: 77.60 % vs Negative Binomial Model 2: 77.36 %) (Supplementary Table S9).

For counties, the CART method based on Random Forest Model 1 provided the highest accuracy at 83.33 % (IQR: 62.83–90.99 %), followed by Random Forest Model 2 (81.17 %, IQR: 68.55–91.33 %). However, for 2021 data, Random Forest Model 2 provided the highest accuracy at 84.60 % (IQR: 67.61–91.96 %) (Supplementary Table S10).

Although Random Forest Model 1 achieved the highest median accuracy, its wider IQR indicates slightly greater variability in accuracy across different predictions. In contrast, Random Forest Model 2’s higher lower quartile (68.55 %) reflects a more reliable lower bound. Thus, while Random Forest Model 1 demonstrates peak accuracy, Random Forest Model 2 may offer a more stable and dependable prediction range.

This indicates that the Random Forest Model 2, with the imputed hospitalizations from the CART method, consistently showed higher accuracy across analyses.

After imputation, the total number of hospitalizations across 305 ZIP codes and all 46 counties, estimated and observed, is presented in Supplementary Table S11 and S12, respectively. The spatial distribution of hospitalizations in South Carolina and the effectiveness of the estimation framework at the ZIP code and county level using 2021 dataset is presented in Supplementary Fig. S2 and S3, respectively.

### 3.2. Weekly estimates

As the Random Forest model, combined with CART for imputation, yielded more accurate estimations in most of the scenarios, we used this combination for weekly ZIP code and county-level estimation. Table 6 provides the weekly median average percent agreement accuracy of hospitalization estimates across ZIP code and county level.

For ZIP codes with sufficient Prisma Health coverage (123 ZIP codes), the median weekly average percent agreement was 72.29 % (IQR: 63.20–75.62 %). Imputation was applied in ZIP codes with insufficient coverage (194 ZIP codes), and the median accuracy was 55.62 % (IQR: 45.00–63.20 %)). When considering both estimated and imputed values, the overall accuracy for all ZIP codes (317 ZIP codes) was 61.02 % (IQR: 51.17–72.29 %).

We found a similar trend at the county level. In counties with sufficient coverage (28 counties), the median weekly average percent agreement was 76.43 % (IQR: 70.33–81.16 %). For counties requiring imputation due to insufficient coverage (18 counties), the accuracy was 65.75 % (IQR: 51.82–69.78 %). Overall, the median percent agreement across all counties (46 counties) was 72.64 % (IQR: 66.13–77.69 %).

**Fig. 4.**
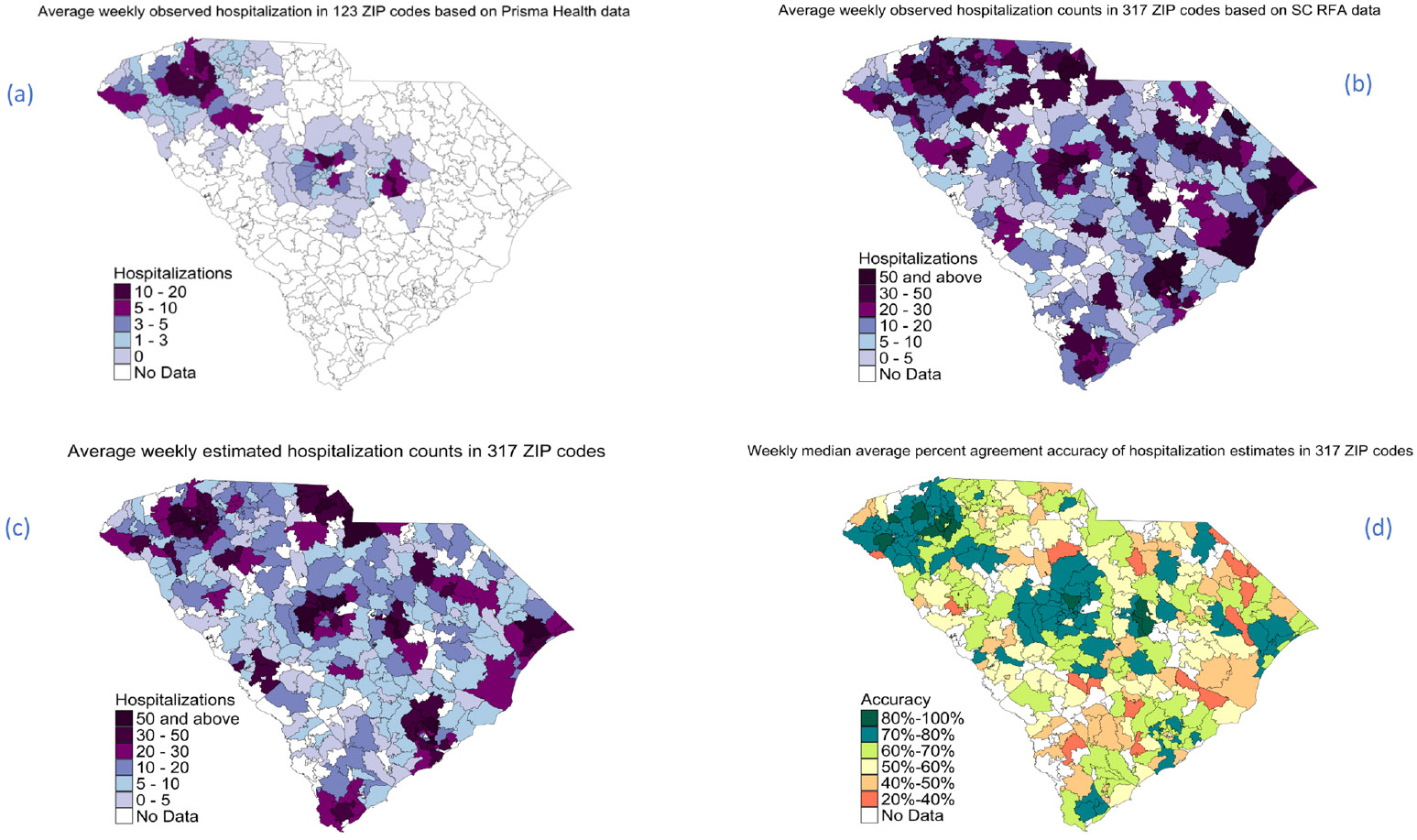
Average weekly observed hospitalization counts based on Prisma Health (a) and SC RFA data (b); average weekly estimated hospitalization counts (c); and weekly median average percent agreement accuracy of observed and estimated hospitalizations (d) at the ZIP code level. No Data implies that data was not available at the state level for testing.

**Fig. 5.**
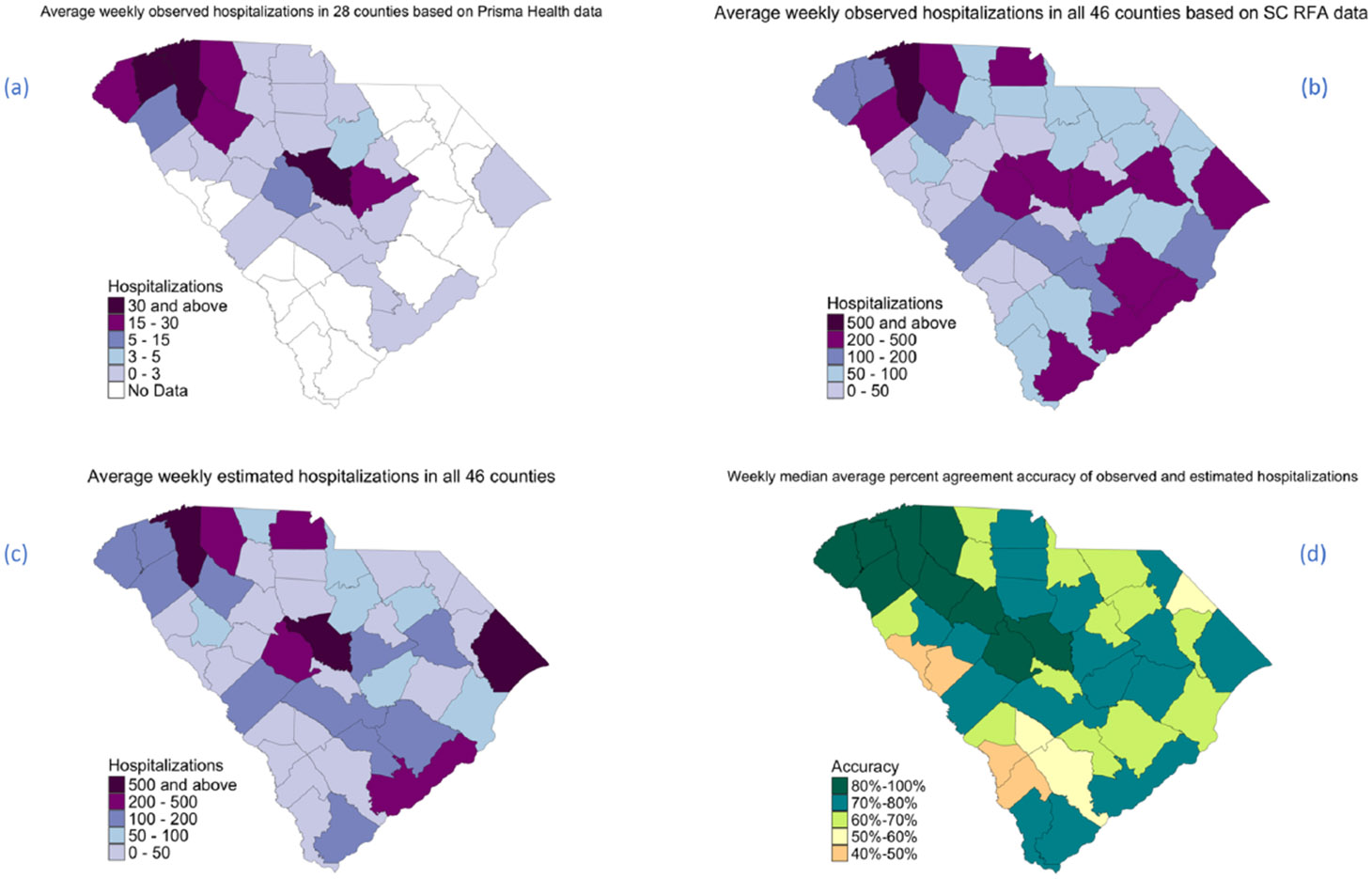
Average weekly observed hospitalization counts based on Prisma Health (a) and SC RFA data (b); average weekly estimated hospitalization counts (c); and weekly median average percent agreement accuracy of observed and estimated hospitalizations (d) at the county level. No Data implies that data was not available at the county level.

Fig. 4 and Fig. 5 illustrate the weekly trends and spatial distribution of hospitalizations across ZIP codes and counties in South Carolina, respectively. It highlights the comparison between observed hospitalizations from Prisma Health data and SC RFA data, as well as the estimated counts generated by our model. The overall trend shows a moderately strong alignment between observed and estimated counts.

## 4. Discussion

The study set out to create a novel estimation framework for COVID-19 hospitalizations at ZIP code and county levels using local health systems and imputing estimates for areas where such data was unavailable. The purpose is to overcome the limitations of relying solely on data from public institutions, which can be delayed due to bureaucratic processes and logistical issues, including data access for researchers. By leveraging more readily available EHR of local health system data, we sought to provide timely and accurate estimations that could inform public health decision-making. However, due to the geographic limitations of health system coverage, it was necessary to impute hospitalization estimates for areas not directly served by Prisma Health. We found that our proposed machine learning approach was highly accurate in estimating current and previous COVID-19 hospitalizations from limited health system data, even in geographic regions lacking sufficient healthcare coverage.

The study delivered estimations at both the ZIP code and county levels, facilitating more targeted and effective public health responses. Additionally, our methodology utilizes existing R packages and was designed to be straightforward, ensuring that the processes of estimation and imputation were not overly complex and can, therefore, be used by those without deep statistical or machine learning backgrounds. Moreover, while this study focused on COVID-19 hospitalizations, the framework is readily adaptable for estimating hospitalizations for other diseases, including influenza and RSV. This would simply require extraction of the relevant ICD-10 codes. However, unlike COVID-19, which resulted in a substantial number of hospitalizations, forecasting accuracy may decrease when estimating and forecasting hospitalizations for other diseases with fewer data points. The findings from this study should also be generalizable to other states that have sufficient health records. This adaptability makes the models helpful in estimating hospitalizations and improving public health responses across diverse regions.

With funding from the Centers for Disease Control and Prevention (CDC) Center for Forecasting and Outbreak Analytics (CFA), we will embed these models into the workflows of South Carolina’s Department of Public Health (SCDPH) and health systems statewide to improve pandemic planning and response. However, in practice, we must continuously refine and retrain these models to adapt to potential changes in inputs. For example, if clinical protocols change (e.g., changes to ICD-10 codes) or there are substantial changes in Census data, then this may alter the relationship between EHR and state health claims data. It is, therefore, worth noting that the primary purpose of this paper is to demonstrate a proof-of-concept for our methodology and does not suppose this is a one-size-fits-all approach. Rather, the primary purpose is to demonstrate that in the absence of quality data, these models obtain reasonable estimates of infectious disease burden in granular geographic areas. Future work will need to adapt and improve these models in other settings (i.e., states or diseases).

### 4.1. Retrospective estimation

Our findings showed that Random Forest models outperformed negative binomial models in estimating hospitalizations at both the ZIP code and county levels. Specifically, Random Forest Model 2, which included additional demographic variables, i.e., sex, age, and race, had consistent median accuracy. This higher accuracy can be attributed to the Random Forest model’s ability to handle nonlinear relationships and predictor interactions more effectively than traditional regression models (Fife and D’Onofrio, 2023). This finding is consistent with previous research indicating that random forests frequently outperform other models in estimating health outcomes (King and Strumpf, 2022; Shakibfar et al., 2023; Song et al., 2022). While neural networks can be robust, they often act as "black boxes," making it difficult to interpret their results. Random Forests, in contrast, offer better interpretability by allowing users to understand the importance of features and decision paths. Moreover, Random Forest is less prone to overfitting than neural networks or gradient-boosting machines because it builds multiple decision trees and averages their predictions. Random Forests have demonstrated superior performance in various studies. For instance, in the study predicting clinically significant prostate cancer, Random Forests outperformed models like GBM and NNs, achieving high accuracy and median percent agreement (Winkel et al., 2020). Similarly, in the agricultural context, RF models showed better predictive accuracy for soil nitrogen and carbon levels than other techniques. This consistent performance across different fields suggests that RF is particularly adaptable to diverse datasets and tasks (Nawar and Mouazen, 2017). However, several studies found that the XGBoost model outperforms Random Forests (Muehlensiepen et al., 2024). Moreover, while they are less prone to overfitting than GBM, Random Forests may still struggle with highly imbalanced data unless techniques like balanced sampling are employed.

For ZIP codes with limited or no Prisma Health coverage, we employed various imputation methods to estimate hospitalizations. The CART method yielded the highest median accuracy. This validates findings from earlier studies that Random Forest models perform very effectively when missing data exists (Batista and Monard, 2003; Cheng et al., 2020).

At the county level, the results closely mirrored those observed at the ZIP code level. Although Random Forest Model 1, which used Prisma Health hospitalization data and subpopulation sizes, achieved the highest median accuracy overall (83.33 %), Random Forest Model 2 outperformed it in specific areas. Model 2 was more accurate for estimating hospitalizations in counties with strong Prisma Health coverage (88.33 % vs 86.61 %) and for imputing hospitalizations in counties with limited Prisma Health data (71.50 % vs 67.43 %). Random Forest Model 2 consistently provided more accurate estimates for estimating and imputing hospitalizations. This consistency makes Random Forest Model 2 the most effective model overall for estimating COVID-19 hospitalizations at the county level.

### 4.2. Weekly nowcasting

We also assessed weekly nowcasting to evaluate the utility of this modeling framework in real-time. The Random Forest Model 2, extended by CART for imputation, demonstrated promising accuracy in estimating weekly COVID-19 hospitalizations across ZIP codes and counties. Areas with sufficient data coverage consistently exhibited high agreement rates, underscoring the model’s robust performance where data availability is reliable. For ZIP codes with adequate coverage, the median weekly accuracy reached 72.29 %, while county-level estimations were slightly higher at 76.43 %. Although the imputed values showed reduced accuracy compared to estimates based on complete data (median accuracy of 55.62 % for ZIP codes and 65.75 % for counties), they still provided a practical approximation where direct estimations were otherwise unattainable. The overall accuracy—61.02 % across ZIP codes and 72.64 % across counties—demonstrates the model’s adaptability across diverse geographic contexts, balancing between robust estimations and necessary imputations. The visual trends in Figs. 4 and 5 reveal a moderately strong alignment between observed and estimated hospitalizations. This alignment suggests the Random Forest model’s effectiveness in capturing weekly hospitalization trends and provides a strong basis for weekly nowcasting.

### 4.3. Implications for public health

The findings of this study have important implications for public health planning and response. The high accuracy of the estimation framework highlights their potential for real-time monitoring and forecasting of COVID-19 hospitalizations. Health officials can gain a comprehensive and timely understanding of hospitalization trends across the state by integrating health system data and using imputation methods in areas where direct data is unavailable. Partnerships with health systems and our methodology based on health system EHR are also beneficial for forecasting as they are robust to policy changes regarding reporting of hospitalizations. For example, on May 1, 2024, the CDC stopped requiring health systems to publish COVID-19 hospitalization data (Carbajal, 2024), which has been a resource for many COVID-19 forecasting efforts nationwide.

#### 4.3.1. Practical application of retrospective estimation (use case)

Analyzing recent infectious disease burden helps plan for future outbreaks and tailor community outreach and intervention programs. For example, Clemson Rural Health (CRH), which owns and operates a fleet of mobile health clinics (MHCs), is using estimates from this model to help identify communities that have been hit hard by respiratory infections over the previous two COVID-19 waves in SC (beginning in August 2024) in order to establish community partnerships in regions where they do not currently service. As a direct use of this modeling framework, CRH is working on developing partnerships in Colleton County. This county experienced a substantial number of hospitalizations and deaths throughout the COVID-19 pandemic, yet lacks sufficient healthcare resources and is, therefore, a prime target for targeted MHC deliveries.

#### 4.3.2. Practical application of nowcasting (use case)

Nowcasting infectious disease hospitalizations is vital for timely public health planning and response. It leverages real-time data to provide an accurate picture of current hospital admissions, enabling health officials to swiftly identify emerging trends and potential surges. This immediate insight supports rapid resource allocation, including staffing, beds, and critical supplies, and informs targeted interventions to prevent further spread. Timely nowcasting facilitates proactive decision-making, ensures optimal healthcare system preparedness, and minimizes adverse outcomes by addressing challenges as they arise. In essence, nowcasting empowers public health systems to act quickly and effectively amid dynamic infectious disease threats. The CDC CFA is currently funding the use cases for this modeling framework. The first, currently underway, is embedding these models into the workflows of CRH and Prisma Health (SC’s largest not-for-profit healthcare provider) to allocate MHCs for infectious disease screening to high-risk areas based on COVID-19 nowcasts, alternative healthcare availability, and establishment of community partnerships. The second use case is to inform health system staffing and hospital bed capacity during infectious disease surges (expected completion in Fall 2025). Our partner health systems for this use case include Prisma Health and the Medical University of South Carolina (MUSC), which combined serve approximately 50 % of SC’s population with coverage in the majority of SC counties.

### 4.4. Limitations and future research

This study has several limitations, too. First and foremost, this study focused on retroactively estimating the number of statewide hospitalizations from health system data. Future research must focus on expanding this modeling framework for real-time prediction. Second, Prisma Health is a large health system that covers nearly 25 % of the SC population and has a presence in half of SC counties. Estimation accuracy may be reduced if based on health systems that serve fewer regions. Also, in practice, these models must be continuously refined to account for changes in inputs (e.g., clinical protocols), which can impact the relationship between EHR and state health claims data. Future work should focus on adapting to evolving data structures and extending the approach to other states or diseases. As a result, retraining may be necessary for different health systems, regions, or new waves of the disease. Third, the data obtained from the SC RFA only provided the month of hospitalizations (and not the exact data). This lack of detailed information could affect the precision of the model, particularly in understanding trends and changes over time. Moreover, the SC RFA data was only available until December 2023, which limited the ability to estimate hospitalizations related to newer COVID-19 variants. Future models should incorporate more recent data to enhance their performance and adapt to emerging variants and trends.

While observed and estimated COVID-19 hospitalization counts align for most areas, notable differences arise in regions with higher observed hospitalization counts. The increased variability and complexity of factors influencing hospitalizations in these regions likely necessitate additional variables or considerations in the model to improve prediction accuracy. In addition, due to the exclusion of ZIP codes with no hospitalization data and/or census data throughout the study period, caution should be taken when extrapolating the study’s findings to such regions. Future research should address these limitations by expanding the geographic scope of the study, incorporating data from multiple health systems, and ensuring the continuous updating of data to include recent developments. Integrating more detailed and high-quality data sources could further refine the model’s accuracy and applicability. This approach could also be adapted to forecast hospitalizations for other diseases or health events, providing a valuable tool for public health officials in various contexts.

### 4.5. Conclusion

This study developed and validated a framework for estimating COVID-19 hospitalizations using health system data and advanced imputation techniques. Our approach demonstrated that Random Forest models, combined with CART for imputation, can accurately estimate hospitalizations at a granular level in real-time, even in regions with limited or no direct data. This methodology is particularly valuable when electronic health records are the primary real-time data source available to modelers. Importantly, our framework not only provides reasonable estimates in areas with partial data but also extends to regions without any data, enhancing public health preparedness. By leveraging these techniques, health officials can improve response efforts, optimize resource allocation, and deploy targeted interventions in high-risk communities. Future research should explore adapting this approach to other infectious diseases and integrating additional real-time data sources to further refine accuracy and applicability.

## Supplementary Material

Supplementary Material

## Figures and Tables

**Table 1:** Demographic characteristics of the hospitalized participants for selected 125 ZIP codes (total hospitalization count > 50 by each ZIP code).

Factors		Data Source	
		Prisma Health	SC RFA
Sex, N (%)			
	Female	11,949 (59.96)	207,814 (56.97)
	Male	7980 (40.04)	156,969 (43.03)
Age in years, N (%)			
	< 18	4204 (21.09)	90,152 (24.71)
	18–44	4814 (24.16)	105,172 (28.83)
	45–64	4674 (23.45)	72,638 (19.91)
	65 and above	6237 (31.30)	96,821 (26.54)
Race, N (%)			
	White	12,803 (64.24)	186,834 (51.22)
Total	Non-white	7126 (35.76)	177,949 (48.78)
		19,929	364,783

**Table 2:** Demographic characteristics of the hospitalized participants for selected 19 counties (total hospitalization count > 100 by each county).

Factors		Data Source	
		Prisma Health	SC RFA
Sex, N (%)			
	Female	12,045 (59.98)	104,230 (56.85)
	Male	8038 (40.02)	79,126 (43.15)
Age in years, N (%)			
	< 18	4247 (21.15)	43,948 (23.97)
	18–44	4849 (24.14)	52,183 (28.46)
	45–64	4708 (23.44)	37,199 (20.29)
	65 and above	6279 (31.27)	50,026 (27.28)
Race, N (%)			
	White	12,884 (64.15)	97,869 (53.38)
Total	Non-white	7199 (35.85)	85,487 (46.62)
		20,083	183,356

**Table 3:** The median percent agreement accuracy ($A_{i}$) of estimated cumulative hospitalizations over the previous 6 months (Jul-Dec ’23) in 125 ZIP codes and 19 counties with sufficient Prisma Health coverage (defined by at least 50 COVID-19 hospitalization encounters per ZIP code and 100 hospitalizations per county between 2020 and 2023). IQR = interquartile range.

Area	$A_{i}A_{i}$ , Median (IQR)						
	Negative Binomial Models					Random Forest Models	
	Model 1	Model 2	Model 3	Model 4	Model 5	Model 1	Model 2
ZIP codes	84.46 % (78.70 % - 92.07 %)	**85.44 % (78.19 % - 91.74 %)**	84.67 % (77.51 % - 91.58 %)	82.23 % (70.30 % - 90.32 %)	77.92 % (70.95 % - 88.51 %)	82.91 % (69.21 % - 90.03 %)	85.02 % (76.15 % - 93.17 %)
County	86.18 % (81.12 % - 91.92 %)	83.01 % (78.75 % - 89.57 %)	81.14 % (78.45 % - 88.22 %)	83.53 % (72.11 % - 87.43 %)	78.09 % (73.71 % - 87.20 %)	86.61 % (80.70 % - 93.11 %)	**88.33 % (81.94 % - 93.01 %)**

**Table 4:** The median percent agreement accuracy ($A_{i}$) of imputed cumulative hospitalizations over the previous 6 months (Jul–Dec ’23) in 180 ZIP codes and 27 counties with insufficient Prisma Health coverage (defined by at least 50 COVID-19 hospitalization encounters per ZIP code and 100 hospitalizations per county between 2020 and 2023). IQR = interquartile range.

Area	$A_{i}$, Median (IQR)				
	Estimated values	Random Forest Algorithm	Predictive Mean Matching	Classification and Regression Trees	Lasso Linear Regression
ZIP codes	Negative Binomial Model 2	59.02 % (40.98– 79.21 %)	61.71 % (42.52–80.71 %)	71.35 % (56.66–86.57 %)	49.10 % (30.46–73.00 %)
	Random Forest Model 2	66.26 % (46.05– 78.69 %)	63.09 % (44.35– 82.77 %)	**77.13 % (60.91–86.22 %)**	55.61 % (33.21–71.41 %)
County	Random Forest Model 1	52.45 % (29.37– 73.97 %)	26.85 % (12.64– 45.45 %)	67.43 % (53.79–88.56 %)	60.78 % (27.03–78.26 %)
	Random Forest Model 2	52.40 % (28.58–74.27 %)	26.07 % (11.90– 44.35 %)	**71.50 % (53.21–85.09 %)**	66.18 % (47.67– 80.77 %)

**Table 5:** The median percent agreement accuracy ($A_{i}$) of cumulative hospitalization estimates over the previous 6 months (Jul-Dec ’23) in 305 ZIP codes and 46 counties. IQR = interquartile range.

Area	$A_{i}$, Median (IQR)				
	Estimated values	Random Forest Algorithm	Predictive Mean Matching	Classification and Regression Trees	Lasso Linear Regression
ZIP codes	Negative Binomial Model 2	76.62 % (54.24–87.98 %)	76.99 % (55.55– 88.04 %)	79.55 % (66.86–89.89 %)	73.27 % (41.91–87.29 %)
	Random Forest Model 2	74.98 % (58.18– 87.70 %)	76.69 % (56.08– 89.85 %)	**80.98 % (68.99–89.66 %)**	70.87 % (47.75– 88.35 %)
County	Random Forest Model 1	75.85 % (42.78–91.83 %)	60.52 % (23.41– 86.27 %)	**83.33 % (62.83–90.99 %)**	78.78 % (59.88– 90.45 %)
	Random Forest Model 2	75.60 % (42.37– 88.90 %)	61.49 % (22.84– 88.14 %)	81.17 % (68.55–91.33 %)	77.64 % (62.57– 90.29 %)

**Table 6:** Percent agreement accuracy ($A_{i}$) of estimated hospitalizations over the previous 7 days in ZIP codes and counties between July 3 and Oct 1, ’23. Average percent agreement computed for each zip code and county. Results are presented as the median (average) percent agreement and IQR.

Area	Average $A_{i}$, Median (IQR)		
	Sufficient Prisma Health coverage	Insufficient Prisma Health coverage	Overall estimates
ZIP codes	72.29 % (63.20–75.62 %)	55.62 % (45.00–63.20 %)	61.02 % (51.17–72.29 %)
County	76.43 % (70.33–81.16 %)	65.75 % (51.82–69.78 %)	72.64 % (66.13–77.69 %)

## Data Availability

The authors do not have permission to share data.

## Appendix A. Supporting information

Supplementary data associated with this article can be found in the online version at doi:10.1016/j.epidem.2025.100823.
