# Supplementary material for: Machine learning approaches for real-time ZIP code and county-level estimation of state-wide infectious disease hospitalizations using local health system data
Source: Epidemics. Author manuscript; Available in PMC 2025 Dec 6. (PMC12681050; doi:10.1016/j.epidem.2025.100823)
Supplement: Supplementary Material [file NIHMS2116305-supplement-Supplementary_Material.docx]

Supplementary Tables

Tables S1: ICD-10 codes for COVID-19 hospitalization obtain from Prisma Health.

| Code | Description |
| --- | --- |
| U07.1 | COVID-19, virus identified: This code is used to report cases where the patient has tested positive for COVID-19. It applies to both symptomatic and asymptomatic cases. |
| J12.82 | Pneumonia due to coronavirus disease 2019: This code is used when a patient has pneumonia specifically caused by COVID-19. |
| J06.9 | Acute upper respiratory infection, unspecified: While this code does not specifically indicate COVID-19, it can be used when a patient presents with symptoms of an acute upper respiratory infection without a confirmed diagnosis of COVID-19. |
| Z86.16 | Personal history of COVID-19: This code is used to document a personal history of COVID-19 in cases where the patient has recovered. |
| J80 | Acute respiratory distress syndrome (ARDS): This code can be used when COVID-19 leads to ARDS, a severe respiratory condition. |
| B97.29 | Other coronavirus as the cause of diseases classified elsewhere: This code can be used when COVID-19 is identified as the cause of complications or other diseases such as pneumonia or respiratory distress. |

Tables S2: ICD-10 codes for COVID-19 hospitalization obtain from SC RFA.

| Code | Description |
| --- | --- |
| U07.1 | COVID-19 |
| J12.82 | Pneumonia due to coronavirus disease 2019 |
| U09.9 | Post COVID-19 condition, unspecified |
| Z11.52 | Encounter for screening for COVID-19 |
| J80 | Contact with and (suspected) exposure to COVID-19 |

Supplementary Figure S1: Flowchart for the selected ZIP Codes for each data source used to estimate and impute South Carolina's COVID-19 hospitalizations.

Table S3: The total number of observed and estimated hospitalizations over the previous 6 months (Jul-Dec ’23) in 125 ZIP codes.

| No. | ZIP | Observed hospitalizations (RFA) | Estimated hospitalizations | | | | | | |
| --- | --- | --- | --- | --- | --- | --- | --- | --- | --- |
|  |  |  | Negative Binomial Models | | | | | Random Forest Models | |
|  |  |  | Model 1 | Model 2 | Model 3 | Model 4 | Model 5 | Model 1 | Model 2 |
| 1 | 29006 | 303 | 372 | 358 | 358 | 354 | 357 | 368 | 347 |
| 2 | 29010 | 639 | 624 | 629 | 628 | 476 | 603 | 508 | 568 |
| 3 | 29016 | 685 | 705 | 695 | 703 | 934 | 649 | 989 | 988 |
| 4 | 29020 | 1152 | 920 | 914 | 923 | 754 | 906 | 739 | 894 |
| 5 | 29033 | 458 | 475 | 454 | 455 | 471 | 430 | 521 | 468 |
| 6 | 29036 | 526 | 668 | 654 | 650 | 799 | 576 | 809 | 713 |
| 7 | 29040 | 394 | 363 | 347 | 351 | 376 | 314 | 439 | 386 |
| 8 | 29044 | 231 | 195 | 203 | 200 | 257 | 190 | 253 | 212 |
| 9 | 29045 | 845 | 874 | 837 | 856 | 946 | 774 | 947 | 890 |
| 10 | 29052 | 92 | 84 | 89 | 89 | 104 | 83 | 137 | 97 |
| 11 | 29053 | 939 | 1093 | 1033 | 1033 | 683 | 976 | 745 | 777 |
| 12 | 29054 | 277 | 361 | 346 | 348 | 306 | 343 | 357 | 324 |
| 13 | 29061 | 569 | 578 | 606 | 633 | 553 | 525 | 631 | 612 |
| 14 | 29063 | 1083 | 1309 | 1180 | 1211 | 1335 | 971 | 1241 | 1162 |
| 15 | 29070 | 466 | 581 | 543 | 543 | 524 | 531 | 559 | 559 |
| 16 | 29072 | 1272 | 1828 | 1721 | 1712 | 1813 | 1571 | 1423 | 1629 |
| 17 | 29073 | 1473 | 1830 | 1706 | 1699 | 1389 | 1587 | 1139 | 1392 |
| 18 | 29075 | 70 | 123 | 115 | 116 | 126 | 111 | 155 | 122 |
| 19 | 29078 | 484 | 503 | 483 | 484 | 518 | 470 | 529 | 492 |
| 20 | 29080 | 166 | 148 | 155 | 151 | 156 | 152 | 154 | 134 |
| 21 | 29102 | 1130 | 778 | 807 | 804 | 618 | 815 | 639 | 708 |
| 22 | 29104 | 97 | 90 | 86 | 86 | 78 | 76 | 132 | 85 |
| 23 | 29108 | 615 | 777 | 758 | 761 | 720 | 741 | 730 | 745 |
| 24 | 29115 | 1153 | 943 | 1015 | 1001 | 937 | 1038 | 703 | 979 |
| 25 | 29118 | 532 | 464 | 479 | 474 | 614 | 480 | 580 | 579 |
| 26 | 29123 | 302 | 358 | 331 | 332 | 254 | 314 | 330 | 259 |
| 27 | 29125 | 160 | 171 | 165 | 165 | 151 | 155 | 159 | 151 |
| 28 | 29127 | 191 | 281 | 277 | 277 | 321 | 269 | 389 | 332 |
| 29 | 29128 | 237 | 179 | 183 | 181 | 193 | 172 | 215 | 188 |
| 30 | 29130 | 230 | 222 | 224 | 223 | 296 | 218 | 266 | 256 |
| 31 | 29135 | 342 | 359 | 358 | 354 | 364 | 358 | 328 | 338 |
| 32 | 29138 | 341 | 314 | 308 | 304 | 405 | 307 | 394 | 358 |
| 33 | 29150 | 2619 | 3188 | 2699 | 3575 | 1957 | 1764 | 1779 | 2008 |
| 34 | 29153 | 935 | 795 | 799 | 886 | 698 | 637 | 825 | 732 |
| 35 | 29154 | 1327 | 1353 | 1266 | 1337 | 1136 | 1004 | 1198 | 1179 |
| 36 | 29160 | 296 | 351 | 332 | 334 | 264 | 321 | 329 | 289 |
| 37 | 29168 | 210 | 179 | 162 | 167 | 179 | 140 | 251 | 213 |
| 38 | 29169 | 958 | 1058 | 1034 | 1031 | 743 | 988 | 810 | 886 |
| 39 | 29170 | 729 | 924 | 864 | 870 | 699 | 833 | 686 | 741 |
| 40 | 29172 | 393 | 417 | 398 | 402 | 355 | 386 | 361 | 359 |
| 41 | 29178 | 135 | 173 | 165 | 166 | 154 | 161 | 159 | 147 |
| 42 | 29180 | 576 | 474 | 486 | 479 | 501 | 465 | 552 | 529 |
| 43 | 29201 | 584 | 703 | 694 | 686 | 605 | 651 | 674 | 577 |
| 44 | 29203 | 2694 | 2788 | 2950 | 3994 | 2179 | 1943 | 1568 | 2007 |
| 45 | 29204 | 894 | 760 | 755 | 770 | 734 | 663 | 814 | 760 |
| 46 | 29205 | 568 | 736 | 685 | 686 | 756 | 626 | 751 | 754 |
| 47 | 29206 | 515 | 601 | 573 | 577 | 686 | 516 | 789 | 709 |
| 48 | 29209 | 1215 | 1220 | 1179 | 1265 | 1452 | 970 | 1321 | 1460 |
| 49 | 29210 | 2029 | 2455 | 2190 | 2634 | 1625 | 1621 | 1315 | 1619 |
| 50 | 29212 | 1045 | 1180 | 1108 | 1137 | 1083 | 906 | 1156 | 1017 |
| 51 | 29223 | 2147 | 2079 | 2116 | 2396 | 2089 | 1701 | 1496 | 2043 |
| 52 | 29229 | 1439 | 1577 | 1592 | 1749 | 1814 | 1327 | 1343 | 1715 |
| 53 | 29301 | 1641 | 1150 | 1173 | 1174 | 1122 | 1182 | 863 | 1123 |
| 54 | 29302 | 563 | 469 | 466 | 470 | 593 | 466 | 556 | 562 |
| 55 | 29303 | 1372 | 1070 | 1086 | 1080 | 848 | 1071 | 756 | 955 |
| 56 | 29306 | 1117 | 735 | 747 | 743 | 630 | 744 | 596 | 675 |
| 57 | 29307 | 838 | 613 | 614 | 612 | 646 | 614 | 570 | 657 |
| 58 | 29316 | 1057 | 856 | 830 | 836 | 888 | 795 | 831 | 882 |
| 59 | 29322 | 267 | 216 | 221 | 222 | 325 | 216 | 372 | 313 |
| 60 | 29323 | 690 | 491 | 472 | 472 | 471 | 452 | 478 | 461 |
| 61 | 29325 | 1214 | 991 | 930 | 958 | 653 | 704 | 901 | 838 |
| 62 | 29330 | 471 | 343 | 342 | 346 | 311 | 346 | 353 | 327 |
| 63 | 29332 | 133 | 125 | 115 | 115 | 137 | 106 | 164 | 127 |
| 64 | 29334 | 662 | 560 | 531 | 534 | 550 | 483 | 634 | 608 |
| 65 | 29335 | 276 | 183 | 178 | 179 | 221 | 164 | 262 | 217 |
| 66 | 29341 | 967 | 739 | 723 | 721 | 658 | 715 | 659 | 767 |
| 67 | 29349 | 1260 | 1056 | 1059 | 1056 | 1024 | 1002 | 867 | 970 |
| 68 | 29351 | 113 | 104 | 93 | 95 | 65 | 80 | 141 | 89 |
| 69 | 29356 | 216 | 210 | 214 | 215 | 298 | 201 | 371 | 308 |
| 70 | 29360 | 1797 | 1600 | 1419 | 1524 | 1041 | 990 | 1284 | 1238 |
| 71 | 29365 | 531 | 465 | 443 | 442 | 482 | 377 | 592 | 522 |
| 72 | 29369 | 493 | 446 | 434 | 436 | 528 | 424 | 549 | 496 |
| 73 | 29376 | 329 | 275 | 256 | 258 | 309 | 249 | 333 | 276 |
| 74 | 29379 | 1409 | 1112 | 1117 | 1118 | 611 | 1120 | 645 | 820 |
| 75 | 29384 | 232 | 146 | 138 | 139 | 150 | 121 | 201 | 156 |
| 76 | 29385 | 418 | 325 | 320 | 320 | 307 | 298 | 347 | 299 |
| 77 | 29388 | 710 | 591 | 552 | 550 | 520 | 504 | 546 | 527 |
| 78 | 29601 | 499 | 563 | 530 | 527 | 457 | 475 | 559 | 494 |
| 79 | 29605 | 1818 | 1853 | 1726 | 1864 | 1739 | 1293 | 1657 | 1763 |
| 80 | 29607 | 1524 | 1899 | 1702 | 1723 | 1779 | 1197 | 1744 | 1589 |
| 81 | 29609 | 1140 | 1308 | 1176 | 1149 | 1188 | 847 | 1279 | 1076 |
| 82 | 29611 | 1976 | 1972 | 1736 | 2019 | 1367 | 1261 | 1410 | 1505 |
| 83 | 29615 | 988 | 1066 | 1054 | 1025 | 1465 | 807 | 1378 | 1156 |
| 84 | 29617 | 1351 | 1208 | 1125 | 1134 | 1220 | 809 | 1404 | 1232 |
| 85 | 29621 | 1352 | 1151 | 1084 | 1076 | 1294 | 1062 | 1010 | 1194 |
| 86 | 29624 | 833 | 624 | 639 | 629 | 532 | 632 | 559 | 623 |
| 87 | 29625 | 1040 | 895 | 864 | 854 | 883 | 820 | 813 | 914 |
| 88 | 29626 | 429 | 377 | 367 | 364 | 487 | 359 | 506 | 433 |
| 89 | 29627 | 643 | 527 | 517 | 513 | 556 | 485 | 557 | 596 |
| 90 | 29630 | 470 | 448 | 430 | 430 | 553 | 362 | 692 | 611 |
| 91 | 29631 | 254 | 294 | 284 | 283 | 508 | 245 | 550 | 469 |
| 92 | 29640 | 1957 | 3121 | 2223 | 1966 | 1801 | 1055 | 1714 | 1450 |
| 93 | 29642 | 1225 | 2410 | 1841 | 1752 | 1955 | 821 | 1651 | 1393 |
| 94 | 29643 | 113 | 111 | 107 | 107 | 112 | 90 | 207 | 159 |
| 95 | 29644 | 1174 | 966 | 885 | 871 | 819 | 677 | 1072 | 943 |
| 96 | 29645 | 630 | 615 | 576 | 577 | 442 | 453 | 670 | 558 |
| 97 | 29646 | 1272 | 957 | 967 | 962 | 912 | 972 | 773 | 952 |
| 98 | 29649 | 855 | 782 | 762 | 762 | 855 | 757 | 715 | 834 |
| 99 | 29650 | 1036 | 1153 | 1045 | 1001 | 1474 | 727 | 1325 | 1167 |
| 100 | 29651 | 2029 | 2809 | 2244 | 2130 | 2448 | 1316 | 1935 | 1706 |
| 101 | 29654 | 480 | 342 | 331 | 332 | 343 | 312 | 420 | 344 |
| 102 | 29655 | 295 | 227 | 231 | 231 | 281 | 230 | 345 | 278 |
| 103 | 29657 | 1029 | 954 | 833 | 795 | 663 | 579 | 951 | 763 |
| 104 | 29661 | 434 | 324 | 322 | 317 | 259 | 244 | 443 | 370 |
| 105 | 29662 | 473 | 450 | 438 | 442 | 540 | 360 | 661 | 580 |
| 106 | 29664 | 57 | 54 | 51 | 52 | 77 | 42 | 148 | 91 |
| 107 | 29669 | 535 | 565 | 512 | 499 | 505 | 377 | 783 | 645 |
| 108 | 29670 | 308 | 306 | 283 | 282 | 376 | 247 | 443 | 394 |
| 109 | 29671 | 1442 | 1052 | 979 | 957 | 715 | 763 | 934 | 837 |
| 110 | 29672 | 476 | 498 | 527 | 527 | 562 | 356 | 668 | 564 |
| 111 | 29673 | 1199 | 1459 | 1229 | 1223 | 1293 | 836 | 1474 | 1239 |
| 112 | 29676 | 175 | 190 | 212 | 213 | 259 | 156 | 383 | 314 |
| 113 | 29678 | 1306 | 1092 | 1037 | 1011 | 972 | 778 | 1193 | 1081 |
| 114 | 29680 | 1268 | 1518 | 1275 | 1262 | 1439 | 827 | 1641 | 1350 |
| 115 | 29681 | 1515 | 3107 | 2171 | 1957 | 3259 | 1091 | 2009 | 1758 |
| 116 | 29682 | 227 | 181 | 172 | 172 | 140 | 145 | 270 | 206 |
| 117 | 29686 | 32 | 49 | 42 | 42 | 46 | 34 | 112 | 57 |
| 118 | 29687 | 1528 | 2081 | 1748 | 1685 | 1905 | 1114 | 1789 | 1455 |
| 119 | 29689 | 156 | 128 | 125 | 126 | 154 | 112 | 225 | 167 |
| 120 | 29690 | 1128 | 954 | 842 | 847 | 1170 | 490 | 1152 | 953 |
| 121 | 29691 | 608 | 495 | 474 | 473 | 511 | 340 | 733 | 622 |
| 122 | 29692 | 224 | 167 | 160 | 160 | 200 | 151 | 261 | 207 |
| 123 | 29693 | 846 | 736 | 707 | 716 | 615 | 473 | 842 | 725 |
| 124 | 29696 | 216 | 193 | 187 | 187 | 209 | 141 | 355 | 243 |
| 125 | 29697 | 425 | 385 | 368 | 366 | 516 | 313 | 626 | 554 |

Table S4: The total number of observed and estimated hospitalizations over the previous 6 months (Jul-Dec ’23) in 19 counties.

| County | Observed Hospitalizations (RFA) | Estimated hospitalizations | | | | | | |
| --- | --- | --- | --- | --- | --- | --- | --- | --- |
|  |  | Negative Binomial Models | | | | | Random Forest Models | |
|  |  | Model 1 | Model 2 | Model 3 | Model 4 | Model 5 | Model 1 | Model 2 |
| Aiken | 4157 | 3932 | 3919 | 3868 | 4933 | 4014 | 3864 | 4075 |
| Anderson | 6968 | 5799 | 5634 | 5596 | 5781 | 5227 | 7279 | 6616 |
| Cherokee | 2646 | 2131 | 2064 | 2071 | 2062 | 2062 | 2055 | 2006 |
| Clarendon | 1847 | 1473 | 1450 | 1431 | 1575 | 1442 | 1575 | 1561 |
| Fairfield | 869 | 821 | 838 | 803 | 1220 | 810 | 1025 | 1070 |
| Greenville | 20541 | 34710 | 26993 | 25624 | 28137 | 13743 | 18946 | 18301 |
| Greenwood | 2828 | 2353 | 2305 | 2295 | 2850 | 2328 | 2622 | 2338 |
| Kershaw | 2730 | 2620 | 2550 | 2561 | 2892 | 2512 | 2686 | 2514 |
| Laurens | 4520 | 3540 | 3332 | 3431 | 2497 | 2756 | 3634 | 3822 |
| Lee | 870 | 899 | 1048 | 1003 | 986 | 1065 | 933 | 994 |
| Lexington | 9344 | 10900 | 10472 | 10679 | 7907 | 9868 | 10311 | 10009 |
| Newberry | 1132 | 1578 | 1551 | 1540 | 1900 | 1535 | 1716 | 1709 |
| Oconee | 3893 | 3359 | 3369 | 3385 | 3252 | 2501 | 3924 | 3989 |
| Orangeburg | 3516 | 3129 | 3161 | 3128 | 3527 | 3185 | 2953 | 3106 |
| Pickens | 6492 | 5595 | 5254 | 5104 | 5622 | 3766 | 8014 | 6978 |
| Richland | 15122 | 15886 | 15486 | 20526 | 15106 | 12374 | 14235 | 14061 |
| Spartanburg | 13890 | 11349 | 10970 | 11091 | 8886 | 10353 | 10262 | 10354 |
| Sumter | 6085 | 5252 | 5248 | 5849 | 4711 | 4484 | 5270 | 5514 |
| Union | 1967 | 1757 | 1686 | 1688 | 1371 | 1649 | 1572 | 1548 |

Table S5: The median percent agreement accuracy ($A_{i}$) of estimated hospitalizations over the previous 6 months (Jul-Dec ’21) in 91 ZIP codes (with sufficient Prisma Health coverage as defined by at least 50 COVID-19 hospitalization encounters). IQR = interquartile range.

| $A_{i}$, Median (IQR) | | | | | | |
| --- | --- | --- | --- | --- | --- | --- |
| Negative Binomial Models | | | | | Random Forest Models | |
| Model 1 | Model 2 | Model 3 | Model 4 | Model 5 | Model 1 | Model 2 |
| 82.01% (74.42% - 93.00%) | 83.68% (76.27% - 89.89%) | 84.14% (77.42% - 92.14%) | 80.28% (70.43% - 90.58%) | 70.53% (61.72% - 79.47%) | 82.29% (73.02% - 88.81%) | **86.07% (77.41% - 92.56%)** |

Table S6: The median percent agreement accuracy of estimated hospitalizations in over the previous 6 months (Jul-Dec ’21) 13 counties (where Prisma Health coverage is sufficient).

| $A_{i}$, Median (IQR) | | | | | | |
| --- | --- | --- | --- | --- | --- | --- |
| Negative Binomial Models | | | | | Random Forest Models | |
| Model 1 | Model 2 | Model 3 | Model 4 | Model 5 | Model 1 | Model 2 |
| 89.25% (80.21% - 93.40%) | 85.05% (78.03% - 94.08%) | 89.44% (73.90% - 92.20%) | 82.98% (76.32% - 89.01%) | 72.96% (65.00% - 88.25%) | **89.42% (80.64% - 92.10%)** | 87.79% (83.37% - 93.48%) |

Table S7: The median percent agreement accuracy of imputed hospitalizations over the previous 6 months (Jul-Dec ’21) in 261 ZIP codes (where Prisma Health coverage is insufficient).

| $A_{i}$, Median (IQR) | | | | |
| --- | --- | --- | --- | --- |
| Estimated values | Random Forest Algorithm | Predictive Mean Matching | Classification and Regression Trees | Lasso Linear Regression |
| Negative Binomial Model 2 | 61.94% (44.41% - 79.72%) | 69.43% (54.83% - 85.54%) | 71.88% (58.18% - 86.16%) | 56.47% (34.50% - 74.61%) |
| Random Forest Model 2 | 64.64% (46.62% - 81.27%) | 69.56% (54.87% - 82.75%) | **73.14% (60.82% - 83.95%)** | 57.12% (37.45% - 77.29%) |

Table S8: The median percent agreement accuracy of imputed hospitalizations (Jul-Dec ’21) in 33 counties (where Prisma Health coverage is insufficient).

| $A_{i}$, Median (IQR) | | | | |
| --- | --- | --- | --- | --- |
| Estimated values | Random Forest Algorithm | Predictive Mean Matching | Classification and Regression Trees | Lasso Linear Regression |
| Random Forest Model 1 | 51.16% (35.59% - 68.81%) | 40.87% (20.66% - 53.09%) | 75.38% (50.08% - 94.14%) | 65.83% (42.58% - 82.55%) |
| Random Forest Model 2 | 55.93% (37.39% - 70.54%) | 20.41% (10.82% - 39.78%) | **77.96% (56.59% - 91.88%)** | 55.15% (42.86% - 69.28%) |

Table S9: The median percent agreement accuracy of hospitalization estimates (Jul-Dec ’21) in all 352 ZIP codes.

| $A_{i}$, Median (IQR) | | | | |
| --- | --- | --- | --- | --- |
| Estimated values | Random Forest Algorithm | Predictive Mean Matching | Classification and Regression Trees | Lasso Linear Regression |
| Negative Binomial Model 2 | 72.46% (53.69% - 86.10%) | 77.14% (63.14% - 88.16%) | 77.36% (62.29% - 88.12%) | 68.92% (48.25% - 83.99%) |
| Random Forest Model 2 | 74.32% (55.30% - 87.30%) | 76.07% (62.08% - 88.27%) | **77.60% (65.98% - 87.75%)** | 72.05% (49.06% - 86.06%) |

Table S10: The median percent agreement accuracy of hospitalization estimates (Jul-Dec ’21) in all 46 counties after imputation.

| $A_{i}$, Median (IQR) | | | | |
| --- | --- | --- | --- | --- |
| Estimated values | Random Forest Algorithm | Predictive Mean Matching | Classification and Regression Trees | Lasso Linear Regression |
| Random Forest Model 1 | 66.17% (40.49% - 89.18%) | 53.09% (35.15% - 83.80%) | 80.89% (62.46% - 93.23%) | 78.49% (58.90% - 89.87%) |
| Random Forest Model 2 | 67.72% (44.85% - 87.78%) | 39.78% (16.05% - 85.07%) | **84.60% (67.61% - 91.96%)** | 68.33% (48.64% - 87.78%) |

Table S11: The total number of observed and estimated hospitalizations (Jul-Dec ’23) in 305 ZIP codes after imputation.

| No. | ZIP | Observed hospitalizations (RFA) | Estimated Hospitalizations (Random Forest Model 2) | Imputed Hospitalizations  (using estimates from Random Forest Model 2) | | | |
| --- | --- | --- | --- | --- | --- | --- | --- |
|  |  |  |  | Random Forest Algorithm | Predictive Mean Matching | Classification and Regression Trees | Lasso Linear Regression |
| 1 | 29003 | 363 | NA | 267 | 304 | 269 | 467 |
| 2 | 29006 | 303 | 347 | 347 | 347 | 347 | 347 |
| 3 | 29009 | 95 | NA | 189 | 77 | 100 | 102 |
| 4 | 29010 | 639 | 568 | 568 | 568 | 568 | 568 |
| 5 | 29014 | 66 | NA | 238 | 91 | 91 | -233 |
| 6 | 29015 | 53 | NA | 289 | 166 | 94 | -108 |
| 7 | 29016 | 685 | 988 | 988 | 988 | 988 | 988 |
| 8 | 29018 | 132 | NA | 369 | 281 | 161 | 627 |
| 9 | 29020 | 1152 | 894 | 894 | 894 | 894 | 894 |
| 10 | 29030 | 59 | NA | 219 | 144 | 133 | 39 |
| 11 | 29032 | 236 | NA | 341 | 185 | 260 | 264 |
| 12 | 29033 | 458 | 468 | 468 | 468 | 468 | 468 |
| 13 | 29036 | 526 | 713 | 713 | 713 | 713 | 713 |
| 14 | 29038 | 91 | NA | 183 | 100 | 118 | -110 |
| 15 | 29039 | 152 | NA | 260 | 288 | 147 | 375 |
| 16 | 29040 | 394 | 386 | 386 | 386 | 386 | 386 |
| 17 | 29042 | 364 | NA | 425 | 197 | 277 | 177 |
| 18 | 29044 | 231 | 212 | 212 | 212 | 212 | 212 |
| 19 | 29045 | 845 | 890 | 890 | 890 | 890 | 890 |
| 20 | 29047 | 123 | NA | 216 | 167 | 225 | 218 |
| 21 | 29048 | 231 | NA | 229 | 213 | 227 | 471 |
| 22 | 29052 | 92 | 97 | 97 | 97 | 97 | 97 |
| 23 | 29053 | 939 | 777 | 777 | 777 | 777 | 777 |
| 24 | 29054 | 277 | 324 | 324 | 324 | 324 | 324 |
| 25 | 29055 | 146 | NA | 244 | 213 | 164 | 294 |
| 26 | 29058 | 118 | NA | 338 | 175 | 255 | 212 |
| 27 | 29059 | 258 | NA | 255 | 253 | 220 | 334 |
| 28 | 29061 | 569 | 612 | 612 | 612 | 612 | 612 |
| 29 | 29063 | 1083 | 1162 | 1162 | 1162 | 1162 | 1162 |
| 30 | 29067 | 347 | NA | 455 | 216 | 462 | 312 |
| 31 | 29069 | 215 | NA | 304 | 208 | 188 | 192 |
| 32 | 29070 | 466 | 559 | 559 | 559 | 559 | 559 |
| 33 | 29072 | 1272 | 1629 | 1629 | 1629 | 1629 | 1629 |
| 34 | 29073 | 1473 | 1392 | 1392 | 1392 | 1392 | 1392 |
| 35 | 29075 | 70 | 122 | 122 | 122 | 122 | 122 |
| 36 | 29078 | 484 | 492 | 492 | 492 | 492 | 492 |
| 37 | 29080 | 166 | 134 | 134 | 134 | 134 | 134 |
| 38 | 29101 | 139 | NA | 280 | 177 | 133 | 123 |
| 39 | 29102 | 1130 | 708 | 708 | 708 | 708 | 708 |
| 40 | 29104 | 97 | 85 | 85 | 85 | 85 | 85 |
| 41 | 29105 | 70 | NA | 227 | 72 | 64 | -254 |
| 42 | 29107 | 128 | NA | 307 | 160 | 108 | 274 |
| 43 | 29108 | 615 | 745 | 745 | 745 | 745 | 745 |
| 44 | 29112 | 190 | NA | 180 | 170 | 155 | 271 |
| 45 | 29114 | 114 | NA | 176 | 100 | 71 | -143 |
| 46 | 29115 | 1153 | 979 | 979 | 979 | 979 | 979 |
| 47 | 29118 | 532 | 579 | 579 | 579 | 579 | 579 |
| 48 | 29123 | 302 | 259 | 259 | 259 | 259 | 259 |
| 49 | 29125 | 160 | 151 | 151 | 151 | 151 | 151 |
| 50 | 29127 | 191 | 332 | 332 | 332 | 332 | 332 |
| 51 | 29128 | 237 | 188 | 188 | 188 | 188 | 188 |
| 52 | 29129 | 84 | NA | 267 | 176 | 187 | 108 |
| 53 | 29130 | 230 | 256 | 256 | 256 | 256 | 256 |
| 54 | 29135 | 342 | 338 | 338 | 338 | 338 | 338 |
| 55 | 29137 | 100 | NA | 149 | 125 | 122 | 345 |
| 56 | 29138 | 341 | 358 | 358 | 358 | 358 | 358 |
| 57 | 29142 | 175 | NA | 262 | 328 | 203 | 212 |
| 58 | 29148 | 365 | NA | 269 | 298 | 250 | 422 |
| 59 | 29150 | 2619 | 2008 | 2008 | 2008 | 2008 | 2008 |
| 60 | 29153 | 935 | 732 | 732 | 732 | 732 | 732 |
| 61 | 29154 | 1327 | 1179 | 1179 | 1179 | 1179 | 1179 |
| 62 | 29160 | 296 | 289 | 289 | 289 | 289 | 289 |
| 63 | 29161 | 526 | NA | 532 | 547 | 478 | 802 |
| 64 | 29162 | 125 | NA | 322 | 85 | 151 | 54 |
| 65 | 29163 | 76 | NA | 300 | 80 | 86 | -179 |
| 66 | 29164 | 156 | NA | 267 | 360 | 222 | 429 |
| 67 | 29168 | 210 | 213 | 213 | 213 | 213 | 213 |
| 68 | 29169 | 958 | 886 | 886 | 886 | 886 | 886 |
| 69 | 29170 | 729 | 741 | 741 | 741 | 741 | 741 |
| 70 | 29172 | 393 | 359 | 359 | 359 | 359 | 359 |
| 71 | 29178 | 135 | 147 | 147 | 147 | 147 | 147 |
| 72 | 29180 | 576 | 529 | 529 | 529 | 529 | 529 |
| 73 | 29201 | 584 | 577 | 577 | 577 | 577 | 577 |
| 74 | 29203 | 2694 | 2007 | 2007 | 2007 | 2007 | 2007 |
| 75 | 29204 | 894 | 760 | 760 | 760 | 760 | 760 |
| 76 | 29205 | 568 | 754 | 754 | 754 | 754 | 754 |
| 77 | 29206 | 515 | 709 | 709 | 709 | 709 | 709 |
| 78 | 29209 | 1215 | 1460 | 1460 | 1460 | 1460 | 1460 |
| 79 | 29210 | 2029 | 1619 | 1619 | 1619 | 1619 | 1619 |
| 80 | 29212 | 1045 | 1017 | 1017 | 1017 | 1017 | 1017 |
| 81 | 29223 | 2147 | 2043 | 2043 | 2043 | 2043 | 2043 |
| 82 | 29229 | 1439 | 1715 | 1715 | 1715 | 1715 | 1715 |
| 83 | 29301 | 1641 | 1123 | 1123 | 1123 | 1123 | 1123 |
| 84 | 29302 | 563 | 562 | 562 | 562 | 562 | 562 |
| 85 | 29303 | 1372 | 955 | 955 | 955 | 955 | 955 |
| 86 | 29306 | 1117 | 675 | 675 | 675 | 675 | 675 |
| 87 | 29307 | 838 | 657 | 657 | 657 | 657 | 657 |
| 88 | 29316 | 1057 | 882 | 882 | 882 | 882 | 882 |
| 89 | 29321 | 198 | NA | 292 | 168 | 121 | 223 |
| 90 | 29322 | 267 | 313 | 313 | 313 | 313 | 313 |
| 91 | 29323 | 690 | 461 | 461 | 461 | 461 | 461 |
| 92 | 29325 | 1214 | 838 | 838 | 838 | 838 | 838 |
| 93 | 29330 | 471 | 327 | 327 | 327 | 327 | 327 |
| 94 | 29332 | 133 | 127 | 127 | 127 | 127 | 127 |
| 95 | 29334 | 662 | 608 | 608 | 608 | 608 | 608 |
| 96 | 29335 | 276 | 217 | 217 | 217 | 217 | 217 |
| 97 | 29340 | 1221 | NA | 841 | 835 | 780 | 792 |
| 98 | 29341 | 967 | 767 | 767 | 767 | 767 | 767 |
| 99 | 29349 | 1260 | 970 | 970 | 970 | 970 | 970 |
| 100 | 29351 | 113 | 89 | 89 | 89 | 89 | 89 |
| 101 | 29353 | 213 | NA | 253 | 268 | 177 | 392 |
| 102 | 29356 | 216 | 308 | 308 | 308 | 308 | 308 |
| 103 | 29360 | 1797 | 1238 | 1238 | 1238 | 1238 | 1238 |
| 104 | 29365 | 531 | 522 | 522 | 522 | 522 | 522 |
| 105 | 29369 | 493 | 496 | 496 | 496 | 496 | 496 |
| 106 | 29372 | 242 | NA | 276 | 236 | 201 | 336 |
| 107 | 29374 | 89 | NA | 189 | 148 | 146 | -56 |
| 108 | 29376 | 329 | 276 | 276 | 276 | 276 | 276 |
| 109 | 29379 | 1409 | 820 | 820 | 820 | 820 | 820 |
| 110 | 29384 | 232 | 156 | 156 | 156 | 156 | 156 |
| 111 | 29385 | 418 | 299 | 299 | 299 | 299 | 299 |
| 112 | 29388 | 710 | 527 | 527 | 527 | 527 | 527 |
| 113 | 29401 | 128 | NA | 434 | 370 | 458 | 668 |
| 114 | 29403 | 597 | NA | 853 | 988 | 688 | 931 |
| 115 | 29405 | 1189 | NA | 1054 | 1185 | 1255 | 1003 |
| 116 | 29406 | 1486 | NA | 1254 | 1627 | 1541 | 999 |
| 117 | 29407 | 809 | NA | 1223 | 1610 | 1169 | 1173 |
| 118 | 29410 | 440 | NA | 677 | 773 | 637 | 982 |
| 119 | 29412 | 561 | NA | 1210 | 1298 | 1171 | 1007 |
| 120 | 29414 | 645 | NA | 1173 | 1531 | 1140 | 1126 |
| 121 | 29418 | 876 | NA | 881 | 1255 | 859 | 1045 |
| 122 | 29420 | 646 | NA | 934 | 1216 | 860 | 1003 |
| 123 | 29426 | 58 | NA | 180 | 209 | 69 | 41 |
| 124 | 29429 | 141 | NA | 252 | 358 | 181 | 456 |
| 125 | 29431 | 221 | NA | 300 | 350 | 313 | 696 |
| 126 | 29435 | 170 | NA | 408 | 342 | 184 | 497 |
| 127 | 29436 | 220 | NA | 306 | 274 | 197 | 512 |
| 128 | 29437 | 97 | NA | 394 | 257 | 114 | 506 |
| 129 | 29440 | 1806 | NA | 970 | 1317 | 1155 | 1115 |
| 130 | 29445 | 1480 | NA | 1356 | 1723 | 1888 | 1473 |
| 131 | 29446 | 58 | NA | 273 | 162 | 66 | 27 |
| 132 | 29448 | 95 | NA | 398 | 278 | 137 | 318 |
| 133 | 29449 | 237 | NA | 333 | 539 | 307 | 661 |
| 134 | 29450 | 156 | NA | 317 | 287 | 141 | 294 |
| 135 | 29455 | 463 | NA | 775 | 1025 | 937 | 816 |
| 136 | 29456 | 1058 | NA | 1090 | 1157 | 1303 | 1171 |
| 137 | 29458 | 127 | NA | 287 | 234 | 131 | 500 |
| 138 | 29461 | 1561 | NA | 1315 | 1711 | 1448 | 1046 |
| 139 | 29464 | 740 | NA | 1102 | 1277 | 1385 | 1155 |
| 140 | 29466 | 543 | NA | 940 | 1509 | 1254 | 1214 |
| 141 | 29468 | 102 | NA | 175 | 185 | 117 | 19 |
| 142 | 29470 | 136 | NA | 417 | 436 | 200 | 527 |
| 143 | 29472 | 278 | NA | 444 | 475 | 427 | 709 |
| 144 | 29474 | 93 | NA | 161 | 138 | 82 | 305 |
| 145 | 29475 | 115 | NA | 198 | 115 | 111 | 155 |
| 146 | 29477 | 258 | NA | 435 | 389 | 312 | 564 |
| 147 | 29479 | 333 | NA | 501 | 435 | 283 | 497 |
| 148 | 29481 | 71 | NA | 156 | 111 | 82 | 115 |
| 149 | 29483 | 1636 | NA | 1411 | 2019 | 1749 | 1237 |
| 150 | 29485 | 1503 | NA | 1685 | 1816 | 1980 | 1491 |
| 151 | 29486 | 1462 | NA | 1267 | 1450 | 1323 | 1162 |
| 152 | 29487 | 58 | NA | 189 | 127 | 110 | 375 |
| 153 | 29488 | 1144 | NA | 990 | 1047 | 963 | 1293 |
| 154 | 29492 | 248 | NA | 667 | 843 | 598 | 901 |
| 155 | 29501 | 1950 | NA | 1564 | 1841 | 1707 | 1357 |
| 156 | 29505 | 1084 | NA | 1027 | 1372 | 982 | 1132 |
| 157 | 29506 | 1382 | NA | 1087 | 1355 | 1006 | 1184 |
| 158 | 29510 | 661 | NA | 597 | 721 | 431 | 926 |
| 159 | 29511 | 255 | NA | 264 | 268 | 235 | 366 |
| 160 | 29512 | 629 | NA | 734 | 725 | 803 | 988 |
| 161 | 29520 | 838 | NA | 592 | 780 | 612 | 892 |
| 162 | 29526 | 1593 | NA | 1194 | 1607 | 1319 | 1267 |
| 163 | 29527 | 826 | NA | 896 | 1281 | 1126 | 1323 |
| 164 | 29530 | 128 | NA | 193 | 128 | 95 | 134 |
| 165 | 29532 | 1060 | NA | 876 | 1061 | 826 | 1042 |
| 166 | 29536 | 1847 | NA | 785 | 1079 | 691 | 974 |
| 167 | 29540 | 272 | NA | 251 | 329 | 191 | 508 |
| 168 | 29541 | 452 | NA | 568 | 656 | 413 | 767 |
| 169 | 29543 | 63 | NA | 206 | 46 | 48 | -502 |
| 170 | 29544 | 327 | NA | 505 | 382 | 269 | 414 |
| 171 | 29545 | 154 | NA | 299 | 245 | 89 | 318 |
| 172 | 29546 | 172 | NA | 220 | 302 | 142 | 531 |
| 173 | 29547 | 267 | NA | 401 | 223 | 107 | 239 |
| 174 | 29550 | 1571 | NA | 1047 | 1412 | 1214 | 1090 |
| 175 | 29554 | 544 | NA | 410 | 880 | 461 | 716 |
| 176 | 29555 | 263 | NA | 322 | 349 | 253 | 464 |
| 177 | 29556 | 825 | NA | 566 | 1005 | 611 | 761 |
| 178 | 29560 | 798 | NA | 623 | 873 | 569 | 914 |
| 179 | 29563 | 174 | NA | 183 | 303 | 86 | 266 |
| 180 | 29565 | 521 | NA | 429 | 643 | 259 | 735 |
| 181 | 29566 | 928 | NA | 674 | 859 | 856 | 851 |
| 182 | 29568 | 810 | NA | 585 | 947 | 595 | 882 |
| 183 | 29569 | 1444 | NA | 716 | 971 | 734 | 899 |
| 184 | 29570 | 66 | NA | 217 | 299 | 175 | 651 |
| 185 | 29571 | 1152 | NA | 697 | 1039 | 778 | 1149 |
| 186 | 29572 | 337 | NA | 489 | 484 | 510 | 650 |
| 187 | 29574 | 875 | NA | 481 | 632 | 480 | 816 |
| 188 | 29575 | 513 | NA | 566 | 718 | 790 | 913 |
| 189 | 29576 | 899 | NA | 604 | 1076 | 838 | 795 |
| 190 | 29577 | 1314 | NA | 1036 | 1506 | 1102 | 1161 |
| 191 | 29579 | 1514 | NA | 1163 | 1446 | 1185 | 1095 |
| 192 | 29580 | 78 | NA | 209 | 266 | 89 | 204 |
| 193 | 29581 | 288 | NA | 289 | 256 | 226 | 73 |
| 194 | 29582 | 508 | NA | 658 | 832 | 898 | 784 |
| 195 | 29583 | 190 | NA | 424 | 408 | 204 | 595 |
| 196 | 29584 | 134 | NA | 294 | 192 | 99 | 333 |
| 197 | 29585 | 424 | NA | 627 | 640 | 787 | 718 |
| 198 | 29588 | 1417 | NA | 991 | 1443 | 1435 | 1463 |
| 199 | 29590 | 162 | NA | 400 | 237 | 192 | 244 |
| 200 | 29591 | 227 | NA | 401 | 406 | 229 | 659 |
| 201 | 29592 | 101 | NA | 381 | 161 | 40 | 67 |
| 202 | 29596 | 150 | NA | 198 | 193 | 91 | 105 |
| 203 | 29601 | 499 | 494 | 494 | 494 | 494 | 494 |
| 204 | 29605 | 1818 | 1763 | 1763 | 1763 | 1763 | 1763 |
| 205 | 29607 | 1524 | 1589 | 1589 | 1589 | 1589 | 1589 |
| 206 | 29609 | 1140 | 1076 | 1076 | 1076 | 1076 | 1076 |
| 207 | 29611 | 1976 | 1505 | 1505 | 1505 | 1505 | 1505 |
| 208 | 29615 | 988 | 1156 | 1156 | 1156 | 1156 | 1156 |
| 209 | 29617 | 1351 | 1232 | 1232 | 1232 | 1232 | 1232 |
| 210 | 29620 | 727 | NA | 500 | 766 | 561 | 1065 |
| 211 | 29621 | 1352 | 1194 | 1194 | 1194 | 1194 | 1194 |
| 212 | 29624 | 833 | 623 | 623 | 623 | 623 | 623 |
| 213 | 29625 | 1040 | 914 | 914 | 914 | 914 | 914 |
| 214 | 29626 | 429 | 433 | 433 | 433 | 433 | 433 |
| 215 | 29627 | 643 | 596 | 596 | 596 | 596 | 596 |
| 216 | 29628 | 177 | NA | 242 | 364 | 130 | 371 |
| 217 | 29630 | 470 | 611 | 611 | 611 | 611 | 611 |
| 218 | 29631 | 254 | 469 | 469 | 469 | 469 | 469 |
| 219 | 29638 | 139 | NA | 236 | 235 | 212 | 295 |
| 220 | 29640 | 1957 | 1450 | 1450 | 1450 | 1450 | 1450 |
| 221 | 29642 | 1225 | 1393 | 1393 | 1393 | 1393 | 1393 |
| 222 | 29643 | 113 | 159 | 159 | 159 | 159 | 159 |
| 223 | 29644 | 1174 | 943 | 943 | 943 | 943 | 943 |
| 224 | 29645 | 630 | 558 | 558 | 558 | 558 | 558 |
| 225 | 29646 | 1272 | 952 | 952 | 952 | 952 | 952 |
| 226 | 29649 | 855 | 834 | 834 | 834 | 834 | 834 |
| 227 | 29650 | 1036 | 1167 | 1167 | 1167 | 1167 | 1167 |
| 228 | 29651 | 2029 | 1706 | 1706 | 1706 | 1706 | 1706 |
| 229 | 29653 | 190 | NA | 251 | 343 | 230 | 414 |
| 230 | 29654 | 480 | 344 | 344 | 344 | 344 | 344 |
| 231 | 29655 | 295 | 278 | 278 | 278 | 278 | 278 |
| 232 | 29657 | 1029 | 763 | 763 | 763 | 763 | 763 |
| 233 | 29661 | 434 | 370 | 370 | 370 | 370 | 370 |
| 234 | 29662 | 473 | 580 | 580 | 580 | 580 | 580 |
| 235 | 29664 | 57 | 91 | 91 | 91 | 91 | 91 |
| 236 | 29666 | 238 | NA | 283 | 329 | 284 | 350 |
| 237 | 29669 | 535 | 645 | 645 | 645 | 645 | 645 |
| 238 | 29670 | 308 | 394 | 394 | 394 | 394 | 394 |
| 239 | 29671 | 1442 | 837 | 837 | 837 | 837 | 837 |
| 240 | 29672 | 476 | 564 | 564 | 564 | 564 | 564 |
| 241 | 29673 | 1199 | 1239 | 1239 | 1239 | 1239 | 1239 |
| 242 | 29676 | 175 | 314 | 314 | 314 | 314 | 314 |
| 243 | 29678 | 1306 | 1081 | 1081 | 1081 | 1081 | 1081 |
| 244 | 29680 | 1268 | 1350 | 1350 | 1350 | 1350 | 1350 |
| 245 | 29681 | 1515 | 1758 | 1758 | 1758 | 1758 | 1758 |
| 246 | 29682 | 227 | 206 | 206 | 206 | 206 | 206 |
| 247 | 29684 | 159 | NA | 375 | 364 | 249 | 499 |
| 248 | 29687 | 1528 | 1455 | 1455 | 1455 | 1455 | 1455 |
| 249 | 29689 | 156 | 167 | 167 | 167 | 167 | 167 |
| 250 | 29690 | 1128 | 953 | 953 | 953 | 953 | 953 |
| 251 | 29691 | 608 | 622 | 622 | 622 | 622 | 622 |
| 252 | 29692 | 224 | 207 | 207 | 207 | 207 | 207 |
| 253 | 29693 | 846 | 725 | 725 | 725 | 725 | 725 |
| 254 | 29696 | 216 | 243 | 243 | 243 | 243 | 243 |
| 255 | 29697 | 425 | 554 | 554 | 554 | 554 | 554 |
| 256 | 29702 | 411 | NA | 534 | 572 | 333 | 712 |
| 257 | 29706 | 1123 | NA | 974 | 1214 | 937 | 1153 |
| 258 | 29707 | 103 | NA | 840 | 1220 | 1125 | 1130 |
| 259 | 29708 | 600 | NA | 1314 | 1822 | 1236 | 1217 |
| 260 | 29709 | 320 | NA | 305 | 452 | 270 | 702 |
| 261 | 29710 | 334 | NA | 1179 | 1556 | 1234 | 1290 |
| 262 | 29714 | 81 | NA | 300 | 195 | 115 | 289 |
| 263 | 29715 | 831 | NA | 1260 | 1720 | 1234 | 1206 |
| 264 | 29718 | 77 | NA | 345 | 256 | 170 | 422 |
| 265 | 29720 | 1200 | NA | 1448 | 2043 | 2047 | 1277 |
| 266 | 29728 | 97 | NA | 541 | 733 | 401 | 740 |
| 267 | 29730 | 2395 | NA | 1745 | 2594 | 1851 | 1481 |
| 268 | 29732 | 1691 | NA | 1492 | 2220 | 1669 | 1623 |
| 269 | 29742 | 94 | NA | 221 | 259 | 156 | 311 |
| 270 | 29745 | 780 | NA | 982 | 2003 | 1049 | 1135 |
| 271 | 29801 | 929 | NA | 1127 | 1868 | 1178 | 1152 |
| 272 | 29803 | 661 | NA | 1025 | 1818 | 1454 | 1348 |
| 273 | 29805 | 173 | NA | 321 | 484 | 169 | 900 |
| 274 | 29809 | 55 | NA | 379 | 325 | 145 | 322 |
| 275 | 29810 | 162 | NA | 277 | 505 | 187 | 524 |
| 276 | 29812 | 396 | NA | 525 | 823 | 486 | 1025 |
| 277 | 29817 | 241 | NA | 394 | 535 | 222 | 708 |
| 278 | 29824 | 188 | NA | 426 | 432 | 367 | 685 |
| 279 | 29827 | 104 | NA | 369 | 256 | 184 | 407 |
| 280 | 29828 | 54 | NA | 248 | 96 | 44 | -316 |
| 281 | 29829 | 391 | NA | 604 | 774 | 441 | 1141 |
| 282 | 29832 | 177 | NA | 381 | 558 | 213 | 734 |
| 283 | 29835 | 222 | NA | 321 | 501 | 323 | 587 |
| 284 | 29841 | 497 | NA | 1213 | 1533 | 1233 | 1043 |
| 285 | 29842 | 140 | NA | 438 | 577 | 354 | 843 |
| 286 | 29847 | 192 | NA | 371 | 347 | 228 | 535 |
| 287 | 29851 | 299 | NA | 627 | 554 | 347 | 889 |
| 288 | 29853 | 203 | NA | 362 | 613 | 267 | 760 |
| 289 | 29860 | 438 | NA | 642 | 989 | 623 | 983 |
| 290 | 29902 | 363 | NA | 685 | 964 | 678 | 1027 |
| 291 | 29906 | 793 | NA | 1094 | 1677 | 915 | 1242 |
| 292 | 29907 | 248 | NA | 761 | 948 | 639 | 943 |
| 293 | 29909 | 576 | NA | 558 | 850 | 741 | 842 |
| 294 | 29910 | 953 | NA | 1339 | 1729 | 1317 | 1354 |
| 295 | 29918 | 325 | NA | 409 | 458 | 272 | 629 |
| 296 | 29920 | 261 | NA | 504 | 961 | 570 | 1029 |
| 297 | 29924 | 339 | NA | 332 | 631 | 231 | 726 |
| 298 | 29926 | 589 | NA | 894 | 1345 | 1114 | 1243 |
| 299 | 29927 | 409 | NA | 450 | 647 | 529 | 767 |
| 300 | 29928 | 269 | NA | 523 | 727 | 791 | 684 |
| 301 | 29935 | 105 | NA | 274 | 411 | 159 | 518 |
| 302 | 29936 | 680 | NA | 770 | 1428 | 624 | 1192 |
| 303 | 29940 | 121 | NA | 263 | 604 | 211 | 753 |
| 304 | 29944 | 425 | NA | 325 | 448 | 203 | 497 |
| 305 | 29945 | 226 | NA | 276 | 933 | 233 | 680 |

Table S12: The total number of observed and estimated hospitalizations (Jul-Dec ’23) in all counties after imputation.

| County | Observed Hospitalizations (RFA) | Estimated Hospitalizations (Random Forest Model 2) | Imputed Hospitalizations  (using estimates from Random Forest Model 2) | | | |
| --- | --- | --- | --- | --- | --- | --- |
|  |  |  | Random Forest Algorithm | Predictive Mean Matching | Classification and Regression Trees | Lasso Linear Regression |
| Abbeville | 1148 | NA | 2867 | 419 | 1359 | 1315 |
| Aiken | 4157 | 3864 | 3864 | 3864 | 3864 | 3864 |
| Allendale | 286 | NA | 2527 | 469 | 713 | -2350 |
| Anderson | 6968 | 7279 | 7279 | 7279 | 7279 | 7279 |
| Bamberg | 830 | NA | 2822 | 490 | 913 | -362 |
| Barnwell | 796 | NA | 3285 | 453 | 1501 | 1691 |
| Beaufort | 4450 | NA | 7214 | 426 | 9530 | 7372 |
| Berkeley | 7100 | NA | 6640 | 569 | 11893 | 7770 |
| Calhoun | 416 | NA | 3551 | 455 | 866 | 244 |
| Charleston | 9534 | NA | 8804 | 447 | 13307 | 9206 |
| Cherokee | 2646 | 2055 | 2055 | 2055 | 2055 | 2055 |
| Chester | 1560 | NA | 4110 | 461 | 1656 | 2215 |
| Chesterfield | 1728 | NA | 3391 | 445 | 2170 | 2482 |
| Clarendon | 1847 | 1575 | 1575 | 1575 | 1575 | 1575 |
| Colleton | 1779 | NA | 3161 | 391 | 1654 | 2605 |
| Darlington | 3268 | NA | 3116 | 549 | 2679 | 4063 |
| Dillon | 3026 | NA | 3090 | 541 | 1623 | 2327 |
| Dorchester | 4478 | NA | 6712 | 468 | 7875 | 6539 |
| Edgefield | 734 | NA | 3716 | 418 | 1637 | 1981 |
| Fairfield | 869 | 1025 | 1025 | 1025 | 1025 | 1025 |
| Florence | 7158 | NA | 5816 | 474 | 6970 | 6644 |
| Georgetown | 2915 | NA | 3244 | 565 | 2845 | 3324 |
| Greenville | 20541 | 18946 | 18946 | 18946 | 18946 | 18946 |
| Greenwood | 2828 | 2622 | 2622 | 2622 | 2622 | 2622 |
| Hampton | 1640 | NA | 3127 | 478 | 1513 | 2256 |
| Horry | 13184 | NA | 7866 | 442 | 10786 | 7728 |
| Jasper | 1198 | NA | 3563 | 408 | 1448 | 1014 |
| Kershaw | 2730 | 2686 | 2686 | 2686 | 2686 | 2686 |
| Lancaster | 1674 | NA | 5710 | 385 | 4690 | 4487 |
| Laurens | 4520 | 3634 | 3634 | 3634 | 3634 | 3634 |
| Lee | 870 | 933 | 933 | 933 | 933 | 933 |
| Lexington | 9344 | 10311 | 10311 | 10311 | 10311 | 10311 |
| Marion | 2261 | NA | 3060 | 531 | 1330 | 1990 |
| Marlboro | 975 | NA | 2769 | 451 | 1483 | 1739 |
| McCormick | 273 | NA | 2380 | 483 | 889 | -636 |
| Newberry | 1132 | 1716 | 1716 | 1716 | 1716 | 1716 |
| Oconee | 3893 | 3924 | 3924 | 3924 | 3924 | 3924 |
| Orangeburg | 3516 | 2953 | 2953 | 2953 | 2953 | 2953 |
| Pickens | 6492 | 8014 | 8014 | 8014 | 8014 | 8014 |
| Richland | 15122 | 14235 | 14235 | 14235 | 14235 | 14235 |
| Saluda | 452 | NA | 3704 | 414 | 1165 | 788 |
| Spartanburg | 13890 | 10262 | 10262 | 10262 | 10262 | 10262 |
| Sumter | 6085 | 5270 | 5270 | 5270 | 5270 | 5270 |
| Union | 1967 | 1572 | 1572 | 1572 | 1572 | 1572 |
| Williamsburg | 1841 | NA | 2930 | 485 | 1484 | 1980 |
| York | 7099 | NA | 9588 | 415 | 9671 | 8097 |

Supplementary Figure S2: Total observed hospitalization counts based on Prisma Health (a) and SC RFA data (b); total estimated hospitalization counts (c); and percent agreement accuracy of observed and estimated hospitalizations (d) (Jul-Dec ’21) at the ZIP code level. No Data implies that data was not available at the state level for validation.


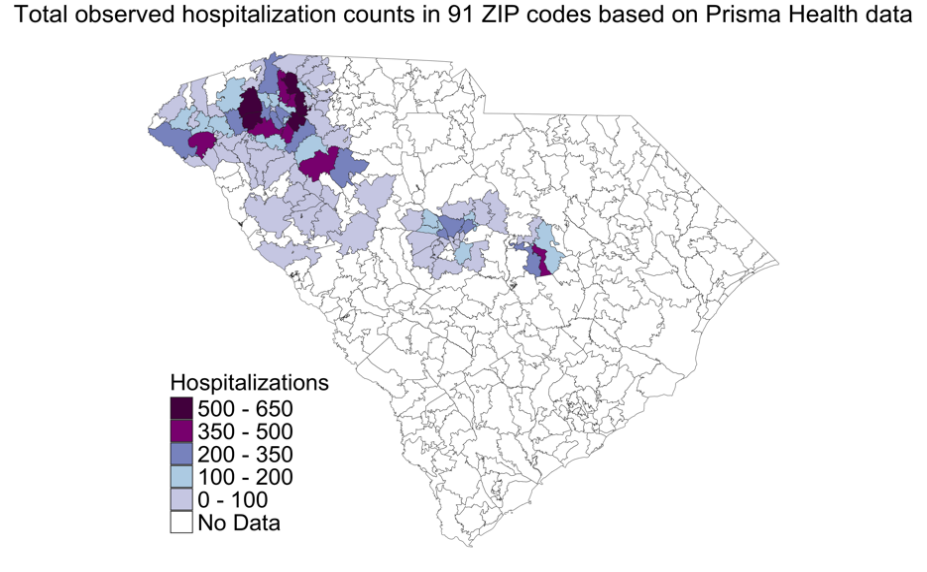

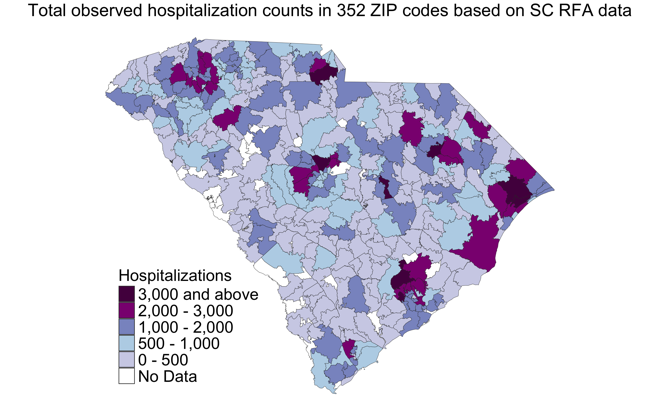

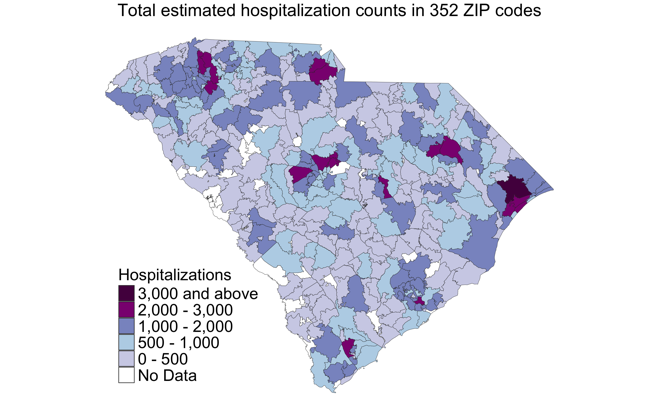

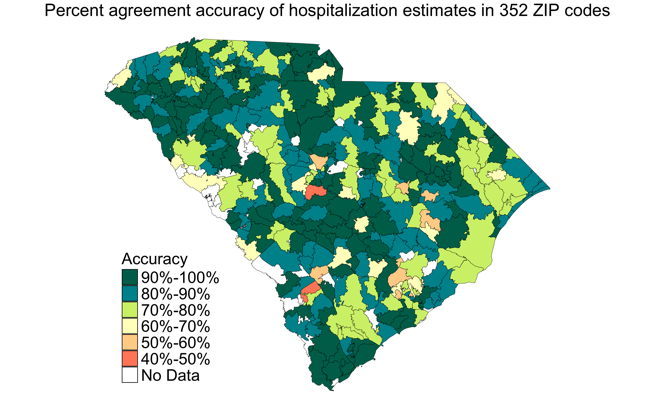


(a)

(c)

(d)

(b)

Supplementary Figure S3: Total observed hospitalizations based on Prisma Health (a) and SC RFA data (b); total estimated hospitalizations (c); and percent agreement accuracy of observed and estimated hospitalizations (d) (Jul-Dec ’21) at the county level.


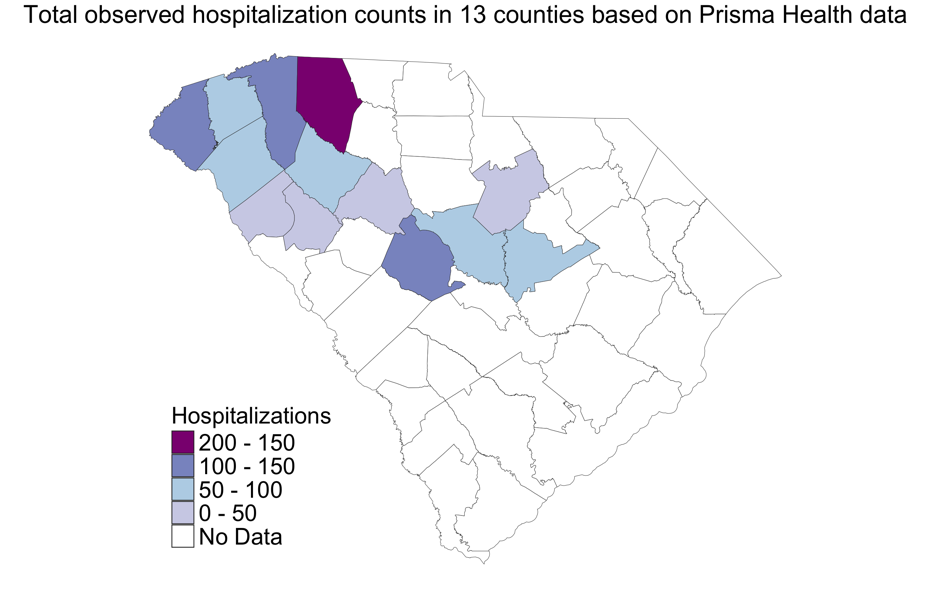

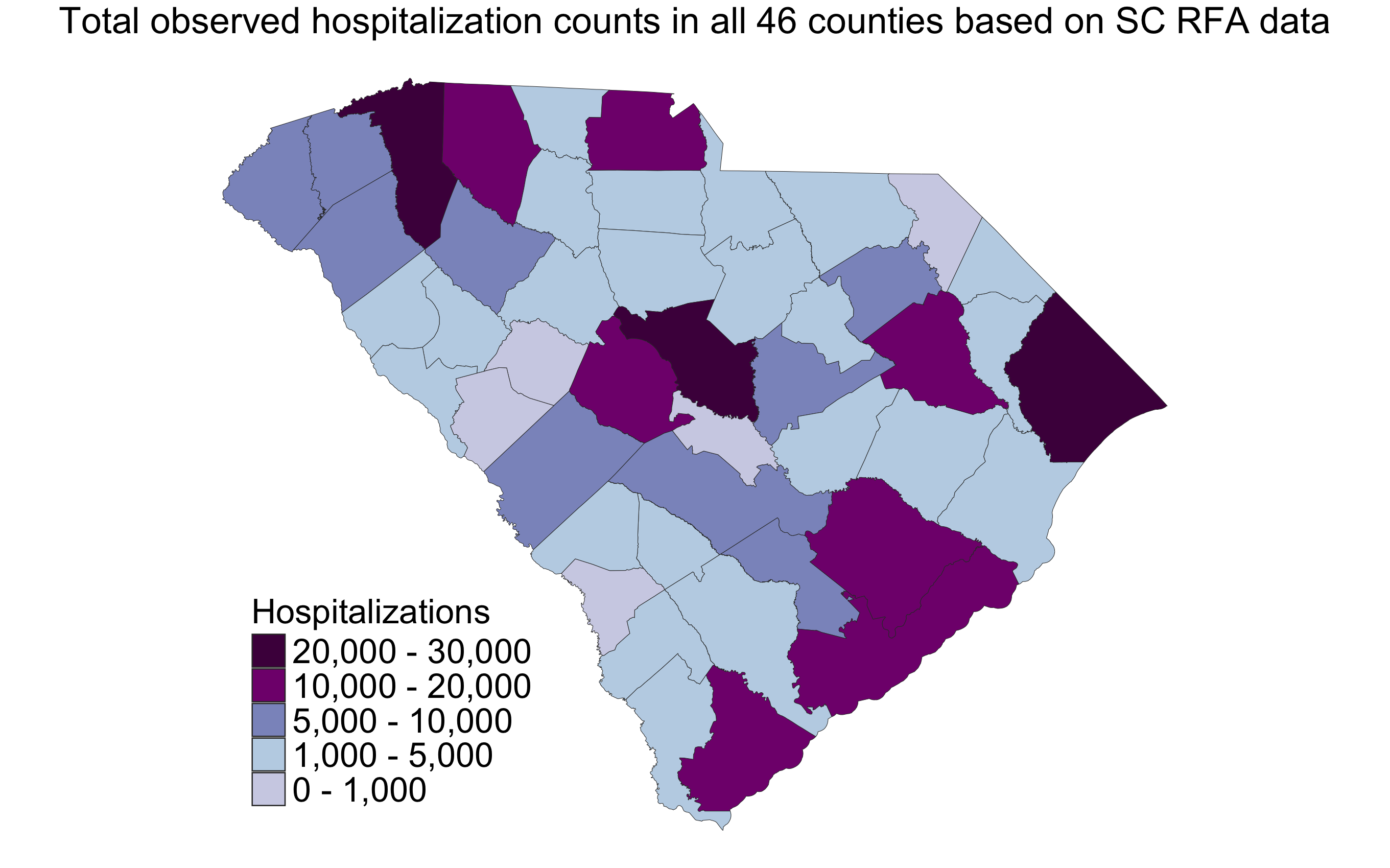

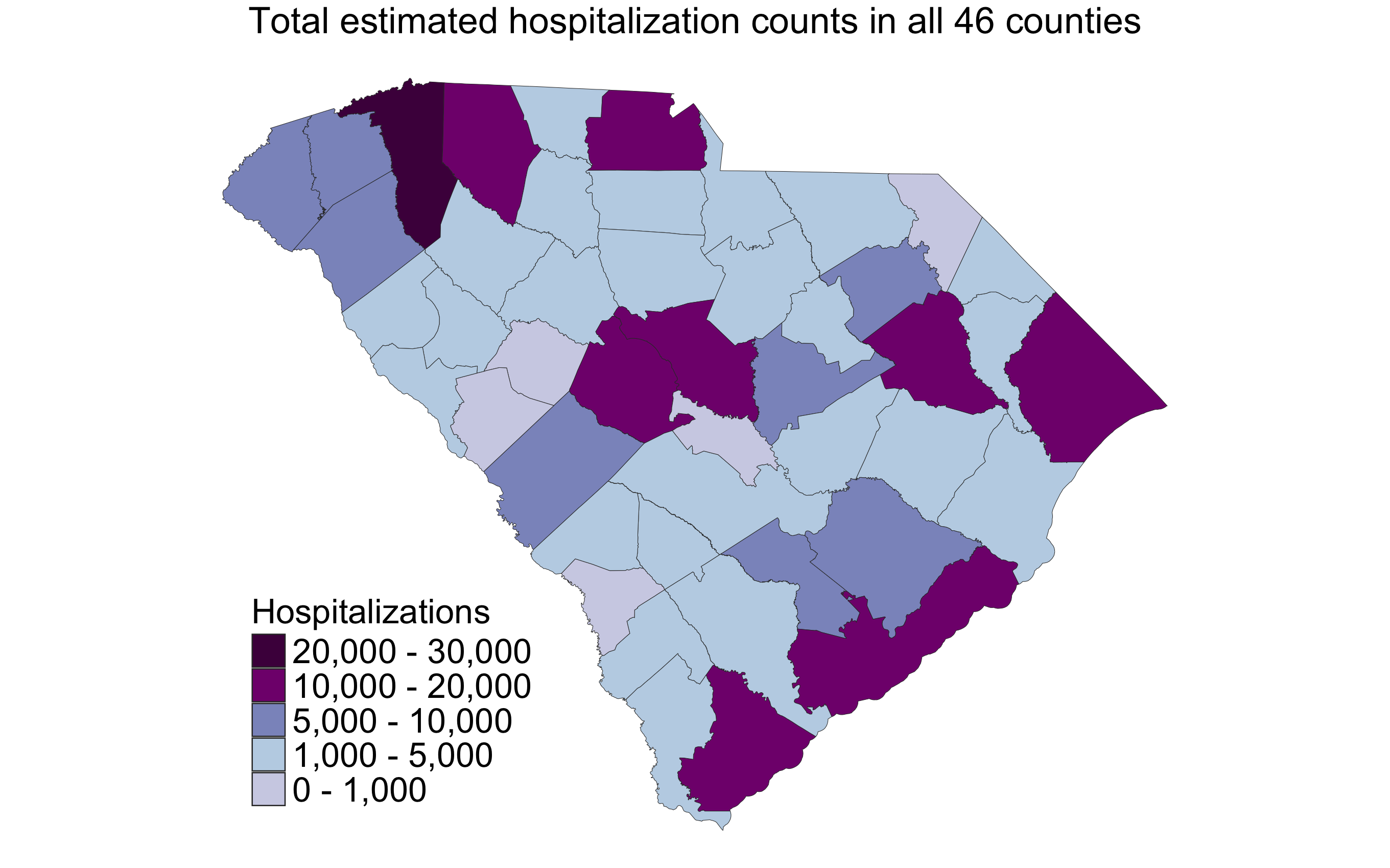

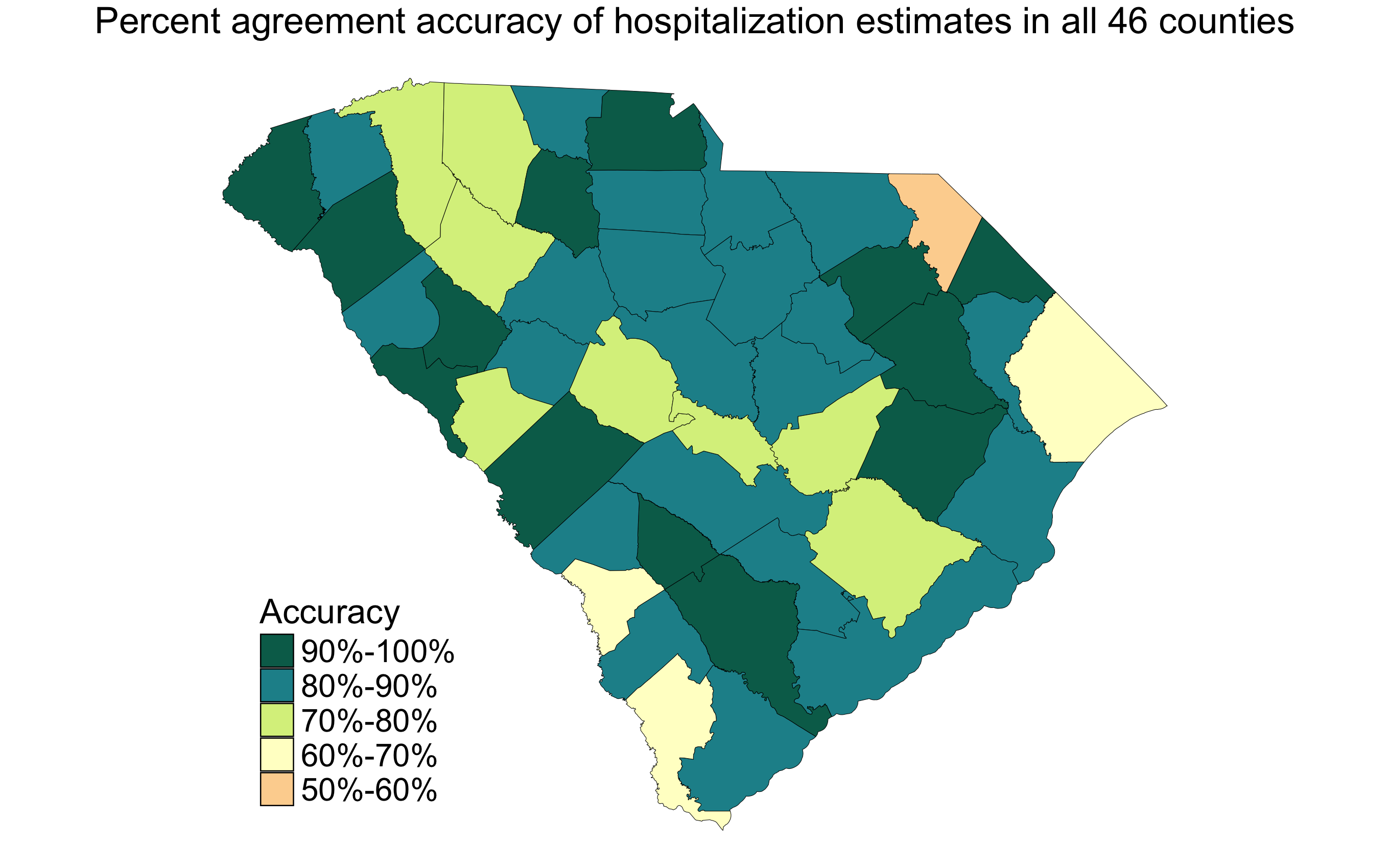


(a)

(c)

(d)

(b)
